# Bulk and single-cell characterisation of the immune heterogeneity of atherosclerosis identifies novel targets for immunotherapy

**DOI:** 10.1186/s12915-023-01540-2

**Published:** 2023-02-28

**Authors:** Jie Xiong, Zhaoyue Li, Hao Tang, Yuchen Duan, Xiaofang Ban, Ke Xu, Yutong Guo, Yingfeng Tu

**Affiliations:** 1 Department of Cardiology, The First Affiliated Hospital of Harbin Medical University, Harbin, 150000 China; 2 Department of Cardiology, The Second Affiliated Hospital of Harbin Medical University, Harbin, 150086 China

**Keywords:** Atherosclerosis, Single-cell RNA sequencing, Immune cells, Heterogeneity, Immunotherapy

## Abstract

**Background:**

Immune cells that infiltrate lesions are important for atherosclerosis progression and immunotherapies. This study was aimed at gaining important new insights into the heterogeneity of these cells by integrating the sequencing results of multiple samples and using an enhanced single-cell sequencing workflow to overcome the limitations of a single study.

**Results:**

Integrative analyses identified 28 distinct subpopulations based on gene expression profiles. Further analysis demonstrated that these cells manifested high heterogeneity at the levels of tissue preferences, genetic perturbations, functional variations, immune dynamics, transcriptional regulators, metabolic changes, and communication patterns. Of the T cells, interferon-induced CD8^+^ T cells were involved in the progression of atherosclerosis. In contrast, proinflammatory CD4^+^ CD28^null^ T cells predicted a poor outcome in atherosclerosis. Notably, we identified two subpopulations of foamy macrophages that exhibit contrasting phenotypes. Among them, TREM2^- ^SPP1^+^ foamy macrophages were preferentially distributed in the hypoxic core of plaques. These glycolytic metabolism-enriched cells, with impaired cholesterol metabolism and robust pro-angiogenic capacity, were phenotypically regulated by CSF1 secreted by co-localised mast cells. Moreover, combined with deconvolution of the bulk datasets, we revealed that these dysfunctional cells had a higher proportion of ruptured and haemorrhagic lesions and were significantly associated with poor atherosclerosis prognoses.

**Conclusions:**

We systematically explored atherosclerotic immune heterogeneity and identified cell populations underlying atherosclerosis progression and poor prognosis, which may be valuable for developing new and precise immunotherapies.

**Supplementary Information:**

The online version contains supplementary material available at 10.1186/s12915-023-01540-2.

## Background

Atherosclerosis, a major cause of cardiovascular disease, is a chronic inflammatory disease caused by the accumulation of cholesterol-containing low-density lipoprotein (LDL) particles under the intima, which leads to the formation of plaques containing lipids, connective tissue, and immune cells [1, 2]. Immunotherapy, the next step in treating cardiovascular diseases (CVDs), can potentially address residual cardiovascular risks beyond the ceiling of benefits that current conventional treatment options provide, such as LDL-cholesterol-lowering regimens and therapies targeting other traditional CVD risk factors [2, 3]. However, the negative results of the Cardiovascular Inflammation Reduction Trial and the increased risks of infection associated with systemic anti-inflammatory treatments (as observed in the Canakinumab Anti-inflammatory Thrombosis Outcomes Study [CANTOS] and Colchicine Cardiovascular Outcomes Trial [COLCOT]) suggest that a one-size-fits-all approach without considering immune heterogeneity is inadequate [4, 5].

Single-cell technologies have proven ideal for studying immune heterogeneity and can be used to identify specific pathogenic cell populations, which is key for advancing drug discovery [6]. The single-cell RNA sequencing (scRNA-seq) datasets, data analysis tools, and various applications continue to grow in scope as sequencing costs continue to fall. Early knowledge of the immune landscape of atherosclerotic lesions gained in previous studies may be insufficient because of the difficulties in obtaining fresh human atherosclerosis samples and limitations in the number of cells and tools used for data analysis [7, 8]. Here, we integrated multiple scRNA-seq datasets for 44,120 immune cells obtained from 17 human atherosclerosis samples. Using a variety of state-of-the-art analytical tools, we identified the tissue preferences, genetic perturbations, functional variations, immune dynamics, transcriptional regulators, metabolic changes, and communication atlas that underlie the heterogeneity of immune cells. Additionally, proinflammatory CD4^+^ CD28^null^ T cells and dysfunctional TREM2^- ^SPP1^+^ foamy macrophages, which were associated with atherosclerosis progression and poor prognosis, were expected to be potential therapeutic targets for future precision medicine.

## Results

### Integrated scRNA-seq analysis quantified the diversity of major cell populations in atherosclerotic lesions

**Fig. 1:**
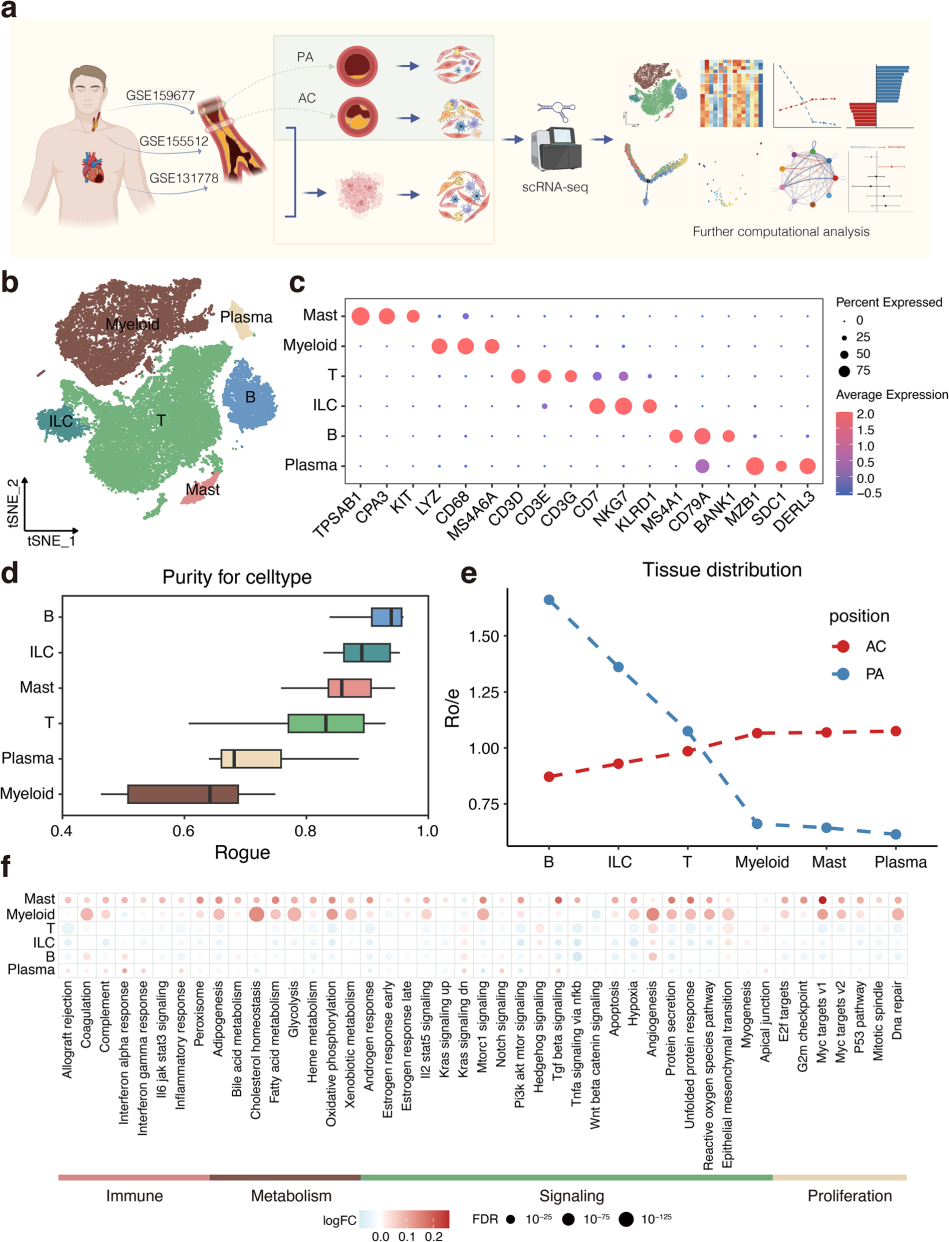
Dissection of the immune landscape in atherosclerosis with scRNA-seq. **a** Schematic of the overall study design. AC, atherosclerotic core; PA, adjacent portion. **b** t-SNE plots showing 44,120 immune cells from atherosclerosis lesions. ILC, Innate lymphoid cells. **c** Dot plot showing average expression of known markers in indicated immune cell types. **d** Boxplot showing cell purity for each cell type by ROGUE. **e** Line chart showing tissue prevalence for each cell type estimated by Ro/e score. **f** Dot plots showing differentially enriched pathways in the global cell type between AC and PA tissues

To understand gene-expression perturbations and generate a comprehensive map of the immune landscape of human atherosclerosis at single-cell resolution, three scRNA-seq datasets were analysed using an integrated bioinformatics method (Fig. 1a). After stringent quality-control filtering and clustering analysis to remove non-immune cells, 44,120 immune cells were further analysed (Additional file 1: Figure S1a-d). These cells were identified as T cells, myeloid cells, B cells, innate lymphoid cells (ILCs), mast cells, and plasma cells based on canonical markers (Fig. 1b, c). Pearson correlation analysis revealed groups of clusters with similar transcriptional profiles that coincided with the main immune cell type (Additional file 1: Figure S1e). In addition, B and mast cells exhibited higher purity, whereas myeloid and plasma cells showed higher heterogeneity (Fig. 1d). Pearson’s correlation analysis also revealed that plasma cells had their own significant and unique transcriptional features when compared with those of B cells and other types of immune cells (Additional file 1: Figure S1e). GSEA revealed that B cells exhibited specific enrichment for cell cycle-related pathways, whereas the plasma population was significantly enriched for biological processes related to protein production and secretion (Additional file 1: Figure S1f).

We quantified the tissue enrichment of all these populations based on Ro/e analysis [9]. Among all populations, mast and myeloid cells were preferentially distributed in the atherosclerotic core (AC), whereas lymphocytes were preferentially located in the adjacent portion (PA) (Fig. 1e). To further investigate variations in the regulatory network of lesion-infiltrating cell subsets, we used hallmark gene sets to assess differences in the pathways of major immune cell populations between the AC and PA. Intriguingly, mast and myeloid cells showed upregulation of a wide variety of pathway activities, including different aspects of immunology, metabolism, signalling, and proliferation (Fig. 1f), implying that distinct anatomical contexts may preferentially remodel these cells and induce specific functional states. Furthermore, inflammatory-response and interferon (IFN)-related pathways were upregulated in AC-filtrating mast cells, and myeloid cells showed greater enrichment for metabolism-related pathways than PA cells (Fig. 1f). The hypoxia-related gene set was also more enriched in mast and myeloid cells from AC samples than in those from PA samples (Fig. 1f). These traits might reflect that immune cell interactions were localised to the hypoxic region of plaques that links mast cells and macrophages, and remodels the AC microenvironment.

### Dissection and clustering of T and ILC cells in atherosclerotic plaques

**Fig. 2:**
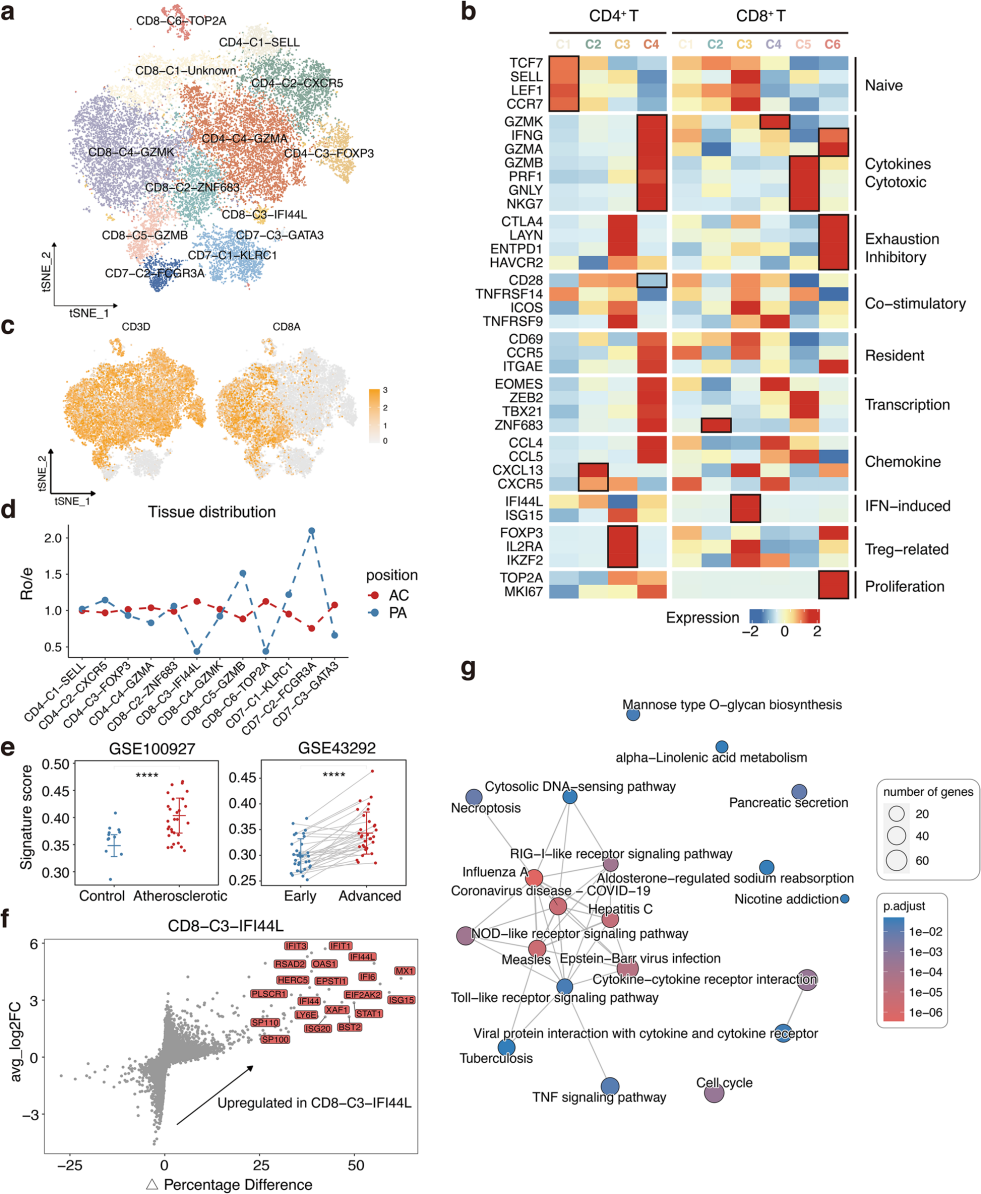
The lineage and characteristics of T and ILC cells in atherosclerotic lesions. **a** t-SNE plot of 24,183 T and ILC cells. **b** Heatmap showing the normalised average expression of selected T cell function-associated genes in each cell subpopulation. **c** Feature plots of canonical marker genes. **d** Line chart showing tissue prevalence for each cell type estimated by Ro/e score. **e** Boxplots showing infiltrating score for CD8-C3-IFI44L subset in atherosclerotic lesions (*n* = 29) and control arteries (*n* = 12) without atherosclerotic lesions (left) and paired early (*n* = 32) and advanced (*n* = 32) lesions (right). ****, *P* ≤ 0.0001. Student’s *t* test (left) and paired Student’s *t* test (right). **f** Volcano plot showing differential gene expression for CD8-C3-IFI44L subset. Genes labelled have log-fold change > 1, Δ Percentage Difference > 30% and adjusted *P*-value from Wilcoxon rank sum test < 0.05. **g** Gene set enrichment analysis of CD8-C3-IFI44L subset

In total, 24,183 cells were clustered into 13 separate subsets, namely four CD4^+^ T cell subsets, six CD8^+^ T cell subsets, and three ILC subsets (Fig. 2a, Additional file 2: Figure S2a). The subpopulations of T lymphocytes that infiltrate atherosclerosis were identified and characterised using the expression of functional markers and marker genes (Fig. 2b, c, Additional file 2: Figure S2b). Based on the Ro/e index, CD8-C5-GZMB T cells was preferentially distributed in the PA, whereas the opposite was true for CD8-C3-IFI44L and CD8-C6-TOP2A T cells (Fig. 2d). To the best of our knowledge, CD8-C3-IFI44L and CD8-C6-TOP2A T cells have not been previously reported in similar studies.

The infiltration of CD8-C3-IFI44L T cells increased with disease progression (Fig. 2e), suggesting that these cells may participate in the development of atherosclerosis. The high expression of IFN-induced genes and enrichment of IFN-related responses observed here suggest that accumulation of type-1 and type-2 IFNs (Fig. 2f, g, Additional file 2: Figure S2c), due to chronic inflammation or viral infections, might have a significant impact on the make-up and functionality of these T cells.

*TOP2A* and *MKI67*, well-established proliferation markers, were highly expressed in the CD8-C6-TOP2A T cells (Fig. 2b). Interestingly, *CTLA4*, *LAYN*, *ENTPD1*, and* HAVCR2* (biomarkers of exhausted T cells) were significantly upregulated in these T cells (Fig. 2b). In addition, *IFNG* and *GZMA*, which are effector molecules, were highly expressed in these cells (Fig. 2b). Taken together, we identified a population of cells expressing both cell proliferation-, cytotoxic-, and exhausted-related genes, with a preference for distribution in the AC.

For the three populations obtained from the ILCs reclustering, we first compared CD7-C1 and CD7-C2 clusters, which were particularly high expression of *KLRF1* that is well-defined NK cell marker (Additional file 2: Figure S2b) [10]. Previous studies have further defined NK cells as CD56^bright^ having an increased capacity of cytokine production and CD56^dim^ NK cells having potently cytotoxic [11]. And phylogenetically, CD56^bright^ NK cells are believed to be the precursors of CD56^dim^ NK cells with a preponderance of evidence supporting this linear progression model [12]. Interestingly, in our study, the cytotoxicity-associated genes, such as *FGFBP2*, *SPON2*, *GZMH*, *GZMB*, *PRF1*, and *GNLY*, were upregulated in C2, suggesting that these cells have a more mature and cytotoxic phenotype (Additional file 2: Figure S2d). In addition, C2 overexpressed the dimNK-related marker *FCGR3A*, while C1 overexpressed the briNK-related marker *XCL1* which has been shown to recruit conventional type 1 DCs to the tumour microenvironment (Additional file 2: Figure S2d) [13]. Therefore, we identified that C1 cluster is briNK and the C2 cluster is dimNK. GSEA analyses also confirmed our results. It is worth noting that Proteasome, Fc gamma R-mediated phagocytosis, and Antigen processing and presentation were upregulated in dimNK, indicating these cells play an essential role in linking the innate and adaptive immune systems. Besides, a number of pathways that determine cell migration, such as cell adhesion molecules (CAMs), focal adhesion, and leukocyte transendothelial migration, were also found (Additional file 2: Figure S2e). For the remaining C3 cluster, these cells expressed the markers of NK cells, but *GATA3* that require for ILC2 cell development were highly expressed, indicating that they might be ILC2 cells (Additional file 2: Figure S2b). Furthermore, Zernecke et al. also found such cells in plaques in mice by meta-analysis [7]. Combined with our study, we suggest that these cells are conserved across species in atherosclerotic lesions.

### Trajectory analysis revealed pathogenic, activated CD4^+^ T populations

To further understand the immune dynamics, the pseudotime developmental trajectory analysis was carried out independently with CD4^+^ and CD8^+^ T cells. The lineage structure of T lymphocytes in the atherosclerotic plaque milieu was inferred by the developmental trajectory, which offered a distinctive picture.

**Fig. 3:**
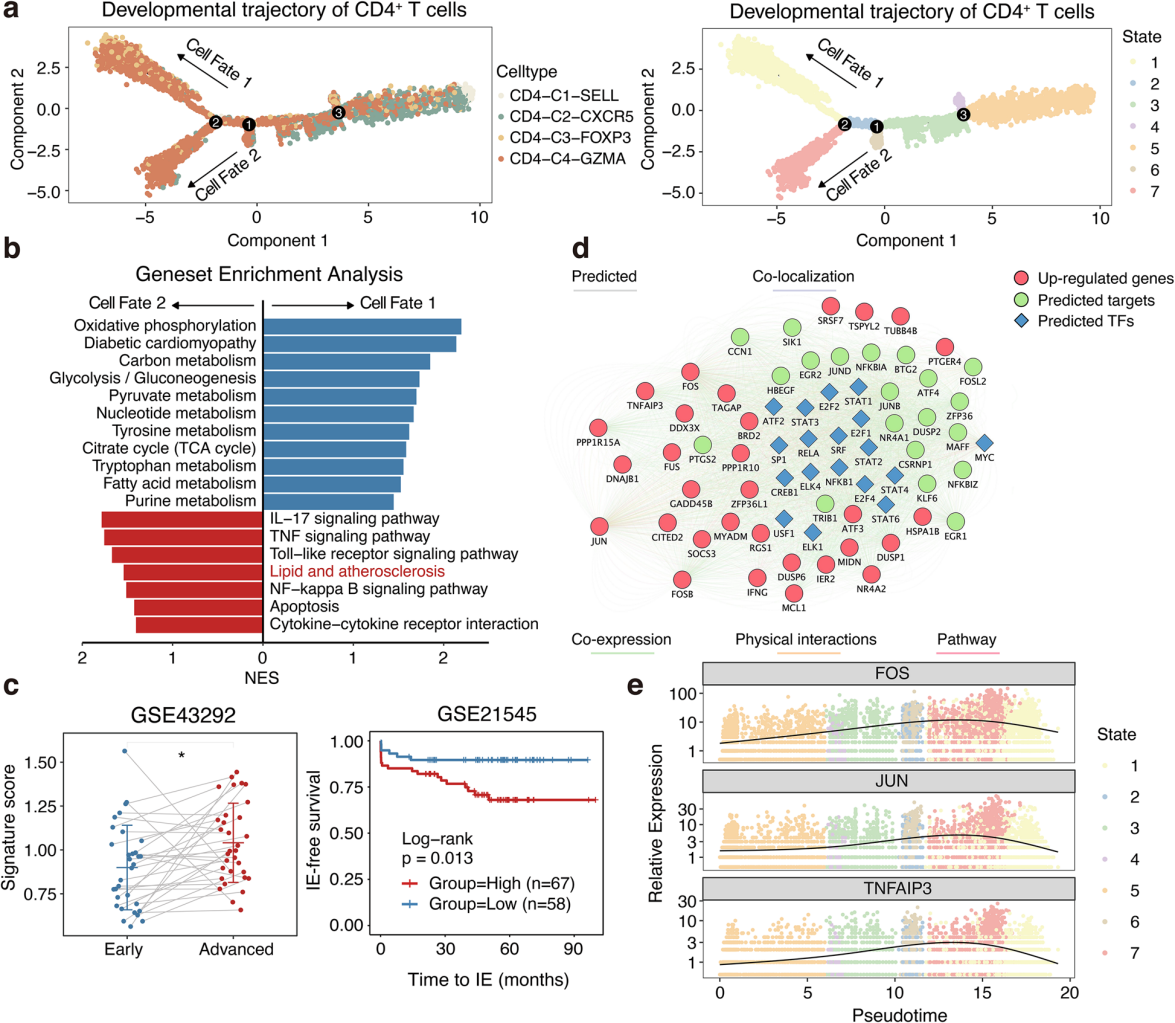
Trajectory analysis of CD4^+^ T populations. **a** The developmental trajectory of CD4^+^ T cells, coloured-coded by the associated cell subpopulations (left) and states (right). **b** Gene set enrichment analysis of the CD4-C4-GZMA population with different cell fates. NES, normalised enrichment score. **c** Boxplots showing infiltrating score of cell fate2 cells in paired early (*n* = 32) and advanced (*n* = 32) lesions (left). Kaplan–Meier survival curve of the ischemic event (IE)–free survival in patients undergoing endarterectomy, stratified high and low according to the mean infiltration score of cell fate2 (right). *, *P* ≤ 0.05. Paired Student’s *t* test (left) and two-sided log-rank test (right). **d** The gene regulatory network constructed by Cytoscape, coloured for the associated gene type (red: top 30 upregulated genes from cell fate2 cells, blue: the upstream transcription factors predicted by RegNetwork, green: the targeted genes predicted by GeneMANIA). **e** Scatter plots showing the expression of selected transcription factors in different cell states as the pseudotime progresses

The trajectory of CD4^+^ T cells showed that CD4-C1-SELL T cells were positioned at the opposite end from CD4-C4-GZMA T cells and that CD4-C2-CXCR5 T and CD4-C3-FOXP3 T cells were mainly located at the centre (Fig. 3a). CytoTRACE also predicted that CD4-C1-SELL T cells have higher differentiation potential and that CD4-C4-GZMA T cells have lower differentiation potential (Additional file 3: Figure S3a). Notably, with pseudotime progression, the CD4-C4-GZMA population differentiated into two distinct branches, indicating the internal heterogeneity of these cells. Thus, we investigated the underlying mechanism of this heterogeneity. Evaluating the expression levels of known pathway members in both CD4-C4-GZMA populations revealed a strong enrichment for metabolism-related pathways for cell fate 1, whereas inflammatory and immune-related pathways were significantly increased in cell fate 2 (Fig. 3b). Strikingly, cell fate 2 additionally exhibited specific enrichment for pathways related to lipids and atherosclerosis (Fig. 3b). We further examined the tissue distributions of these cells in larger pairs of samples. Higher proportions of these cells were observed in advanced plaques (Fig. 3c), supporting the possibility that these cells can accumulate in lesions and play important roles in the progression of atherosclerosis. Furthermore, survival analyses revealed that the high gene-signature scores of these cells significantly predicted the rate of ischemic post-endarterectomy events in an atherosclerotic patient cohort, suggesting that these cells can significantly lower the prognosis of patients with cardiovascular disease (Fig. 3c). In addition, we used RegNetwork to predict upstream TFs for the 30 most upregulated genes in cell fate 2 and Cytoscape to build a regulatory gene network (Fig. 3d). Intriguingly, inflammatory regulators were significantly upregulated in these cells during pseudotime progression (Fig. 3e). Taken together, these results indicate that the presence of these proinflammatory activated T cells may drive the progression of atherosclerotic lesions and lead to the subsequent occurrence of ischemic events.

For CD8^+^ T cells, CytoTRACE and pseudotime analysis also predicted that CD8-C5-GZMB have higher differentiation potential while CD8-C3-IFI44L have lower (Additional file 3: Figure S3b). In order to further understand the state transitions among CD8^+^ T cell subtypes, the functional scores were calculated by AUCell. A descending cytotoxic score and an increasing exhaustion score were also observed concerning CD8-C5-GZMB, CD8-C2-ZNF683, CD8-C4-GZMK, and CD8-C3-IFI44L (Additional file 3: Figure S3c). Remarkably, a lack of tissue-resident markers, such as *CD69*, *CCR5*, and *ITGAE*, was observed in the expression profile of CD8-C5-GZMB (Fig. 2b), which seems to suggest that these cells may have an additional source. In order to probe this question, we further assess the expression patterns of the genes related to migration among CD8^+^ T subsets. And an interesting phenomenon exhibited by CD8-C5-GZMB is that high expression of *CX3CR1* and S1P receptors was observed in these cells (Additional file 3: Figure S3d).

### Dissection and clustering of myeloid cells in atherosclerotic lesions

**Fig. 4:**
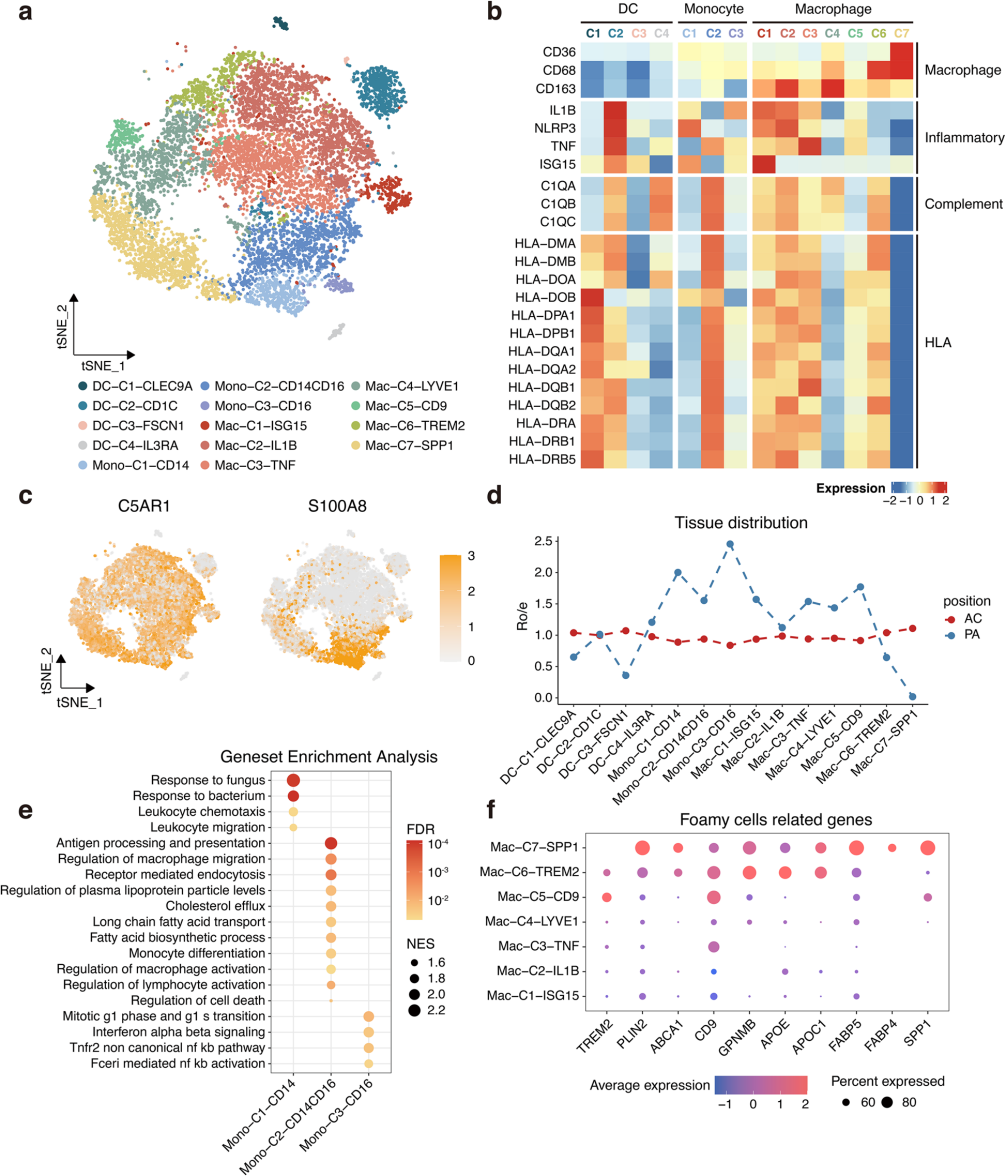
The lineage and characteristics of myeloid cells in atherosclerotic lesions. **a** t-SNE plot of 13,963 myeloid cells. DC, dendritic cells. Mono, monocytes. Mac, macrophages. **b** Heatmap showing the normalised average expression of selected myeloid cells function-associated genes in each cell subpopulation. **c** Feature plots of canonical marker genes. **d** Line chart of tissue prevalence for each cell type estimated by Ro/e score. **e** Gene set enrichment analysis of monocyte subsets. **f** Dot plot showing average expression of foamy cell-related genes in macrophage subsets

Myeloid cells (*n* = 13,963) were the most common population in atherosclerosis. Reclustering revealed 14 clusters: namely DC-C1-CLEC9A (cDC1s), DC-C2-CD1C (cDC2s), DC-C3-FSCN1 (mature DCs), DC-C4-IL3RA (pDCs), Mono-C1-CD14 (classical monocytes), Mono-C2-CD14-CD16 (intermediate monocytes), and Mono-C3-CD16 (non-classical monocytes) cells, as well as seven different macrophage subpopulations (Fig. 4a, Additional file 4: Figure S4a). The expression levels of marker genes and functional signatures were used to identify and characterise the subpopulations of atherosclerosis-infiltrating myeloid cells (Fig. 4b, c, Additional file 4: Figure S4b).

cDC1s and cDC2s showed higher *HLA-DR* gene expression than the remaining DC populations (Fig. 4b). Notably, cDC2 appeared to exhibit broader and more complex functions. The pathways terms, response to interleukin 1, regulation of osteoclast differentiation, and vascular endothelial growth factor production were significantly enriched in cDC2s (Additional file 4: Figure S4c), suggesting that these cells contributed to plaque inflammation, calcification, and neovascularisation, which are key factors in atherosclerosis progression. Mature DCs with high expression of *FSCN1*, *CCR7*, and *LAMP3* were previously identified in atherosclerotic plaques of mice [7]. In this study, cells with low expression of *HLA-DR* and complement-related genes were preferentially distributed in the AC, based on the Ro/e index (Fig. 4b, d). It is noteworthy that the mature DCs closely resembled “mregDCs,” which were recently identified in lung cancer [14]. Importantly, mregDCs comprise a group of DCs that exhibited high expression of maturation molecules, regulatory genes, and migratory markers, as well as TH2 response markers, according to a detailed examination of the signature genes and similarity analysis (Additional file 4: Figure S4d).

By comparing the numbers and distributions of the monocyte subgroups, we found that intermediate monocytes were the most abundant, that classical monocytes were the least abundant, and that all of these subpopulations were preferentially distributed in the PA (Fig. 4a, d). Intriguingly, proinflammatory characteristics were observed in all monocyte subpopulations (Fig. 4b), which prompted us to explore them further. Classic monocytes were predominantly enriched in chemotaxis- and migration-related pathways, whereas non-classical monocytes exhibited a more mature and proinflammatory phenotype (Fig. 4e). However, in addition to exhibiting a stronger antigen-presentation ability, intermediate monocytes also showed a wide range of regulatory effects and enrichment of cholesterol and lipid metabolic pathways (Fig. 4e).

Among the seven subgroups of macrophages identified by reclustering, we found that C1 corresponded to IFINC macrophages; C2 and C3 corresponded to inflammatory macrophages; and C4 corresponded to resident macrophages [8]. In addition, the C5 subset expressed only pro-fibrotic markers such as *TREM2* and *CD9* (Fig. 4f), indicating that these cells comprised a population of pro-fibrotic macrophages [15]. The C6 and C7 subsets, which expressed foam cell-related genes (Fig. 4f), correspond to foam cells [8]. The absence of the SMC-lineage TFs MYOCD and MRTFA in the C6 and C7 subsets, and expression of the myeloid-lineage TFs PU.1 (*SPI1*) and C/EBPß (*CEBPB*) also suggested that all of these cells originated from myeloid cells rather than SMCs (Additional file 4: Figure S4e) [16]. Ro/e analysis revealed that the inflammatory and resident macrophages were preferentially distributed in the PA, whereas the foamy macrophages were preferentially located in the AC (Fig. 4d). Previous data revealed that functional macrophage phenotypes exist across an in vitro M1/M2 dualistic polarisation state [17]. We discovered the co-expression of both M1 and M2 gene signatures in all macrophage subsets (Additional file 4: Figure S4f). Our findings further demonstrate the limitations of such an in vitro polarisation paradigm and point to a more complex macrophage phenotype in vivo.

### Identification of a novel dysfunctional foamy macrophage population and its potential regulatory relationships

**Fig. 5:**
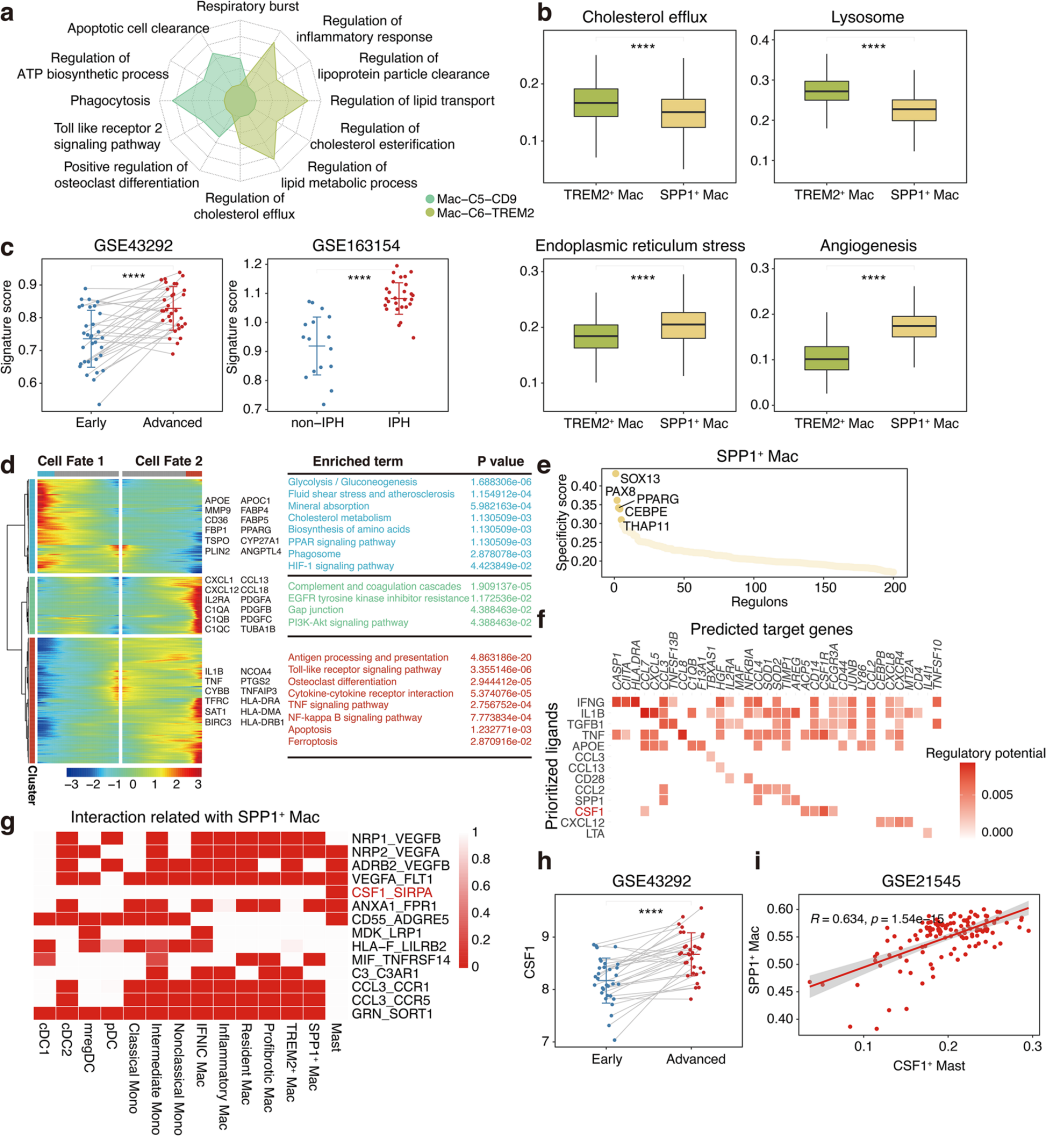
Characterisation of a novel dysfunctional foam macrophage population. **a** Radar plot showing enrichment of GO term for TREM2-related top 30 genes in C5 and C6 macrophage populations. **b** Boxplots showing phenotypic scores of TREM2^+^ Mac and SPP1^+^ Mac. ****, *P* ≤ 0.0001. Wilcoxon rank sum test. **c** Boxplots showing infiltrating score of SPP1^+^ Mac in paired early (*n* = 32) and advanced (*n* = 32) lesions (left) and non-IPH (*n* = 16) and IPH (*n* = 27) lesions. non-IPH, non-intraplaque haemorrhage; IPH, intraplaque haemorrhage. ****, *P* ≤ 0.0001. Wilcoxon rank sum test. **d** Heatmap showing different blocks of DEGs along the pseudotime trajectory (left). Selected KEGG pathways related to corresponding DEGs in heatmap (right). **e** Scatter plot showing the specificity scores of regulons of SPP1^+^ Mac. The top 5 regulons are highlighted. **f** Heatmap showing potential ligands driving the phenotype of SPP1^+^ Mac. **g** Heatmap showing the selected ligand-receptor pairs between SPP1^+^ Mac and other cells in lesions. **h** Boxplots showing *CSF1* expression levels in paired early (*n* = 32) and advanced (*n* = 32) lesions. ****, *P* ≤ 0.0001. Paired Student’s *t* test. **i** Scatter plot showing the Pearson correlation of the proportion of CSF1^+^ mast cells and SPP1^+^ Mac cells

Previous data showed that foamy cells are exclusively TREM2^+^ macrophages and, thus, have been named as TREM2^+^ foamy cells [8, 18, 19]. Unexpectedly, in this study, *TREM2* was highly expressed only in the C6 subset and was expressed at low levels in the C7 subset, which exclusively showed high *SPP1* expression (Fig. 4f). Therefore, to further characterise the diversity between both types of two foam cells, we first examined the TREM2-expression pattern at different lesion sites. Interestingly, *TREM2* was highly expressed in both the PA and AC regions of pro-fibrotic macrophages. In contrast, *TREM2* was highly expressed only in TREM2^+^ SPP1^−^ foamy macrophages at the AC site (Additional file 5: Figure S5a), suggesting that it may be related to the function of foamy cells. Consistently, this finding was verified in the bulk datasets (Additional file 5: Figure S5b). To investigate TREM2-mediated function, we compared the functional annotations of *TREM2* in both groups. Such analysis revealed that several regulatory pathways (lipoprotein particle clearance, cholesterol esterification, and cholesterol efflux) were significantly upregulated in C6 foamy cells (Fig. 5a), suggesting that *TREM2* may help maintain foam cell cholesterol metabolism in the lipid-rich AC. In addition, correlation analysis also confirmed this finding (Additional file 5: Figure S5c). Therefore, given the gene profiles of SPP1^+^ TREM2^-^ foamy macrophages, we hypothesised that these cells may have been dysfunctional.

To test this hypothesis, we computed and compared the classic phenotypic scores of foamy macrophages. The SPP1^+^ macrophages harboured significantly decreased scores related to lysosomes, cholesterol esterification, and cholesterol efflux, but not phagocytosis (Fig. 5b, Additional file 5: Figure S5d). These results illustrate that the abnormal accumulation of lipids and cholesterol in SPP1^+^ macrophages may have been due to imbalanced cholesterol metabolism, which in turn can induce macrophage endoplasmic reticulum stress and death after the formation of necrotic cores, thereby exacerbating the progression of atherosclerosis. Subsequently, we found that SPP1^+^ foamy cells showed significantly higher endoplasmic reticulum stress, apoptosis, and autophagy scores than TREM2^+^ macrophages, confirming our hypothesis (Fig. 5b, Additional file 5: Figure S5d). Intriguingly, we also found that the angiogenesis score was significantly higher in SPP1^+^ macrophages (Fig. 5b). Intimal neovascularisation represents a type of intraplaque haemorrhage that contributes to an increased risk of plaque rupture [20]. To further validate the relationship between SPP1^+^ macrophages and atherosclerosis progression, the gene signatures of SPP1^+^ macrophages were tested using bulk mRNA arrays from isolated macrophage-rich regions of stable and ruptured human atherosclerotic plaques by performing GSEA, which revealed that the gene signatures were significantly upregulated in ruptured plaques (Additional file 5: Figure S5e). In addition, we detected grater infiltration by these cells in advanced atherosclerosis and lesions with intraplaque haemorrhages (Fig. 5c). Taken together, these findings reveal that the unique and dysfunctional SPP1^+^ TREM2^−^ type of foamy cells played an important role in atherosclerosis progression, which has not been elucidated in previous single-cell studies.

To investigate the underlying causes of the SPP1^+^ foamy macrophage phenotype in atherosclerosis, pseudotime developmental trajectory was first performed with monocytes and macrophages to assess potential differentiation relationships. Our results indicate that the trajectory originated from monocytes and bifurcated into distinct developmental pathways, with SPP1^+^ macrophages located at one end of the bifurcations, suggestive of a unique differentiation fate (Additional file 5: Figure S5f). Interestingly, the intermediate monocytes were connected to SPP1^+^ foamy macrophages and showed enrichment of lipid and cholesterol metabolism pathways, indicating that the intermediate monocytes may function as precursor cells for SPP1^+^ foamy macrophages (Fig. 4e, Additional file 5: Figure S5f). Pathway-enrichment analyses showed a strong enrichment for metabolic pathways in cells with different differentiation fates, indicating that metabolic regulation might mediate the phenotypic and functional shift during myeloid differentiation in response to distinct microenvironmental cues (Fig. 5d).

Clearly, the maintenance of cellular phenotypes involves coordinated actions with many regulatory factors, including the regulation of internal cellular TFs and external cell-to-cell communication. Thus, we first discovered a sequence of regulons that underpins SPP1^+^ foamy macrophages using SCENIC analysis [21]. These included the unknown SOX13, PAX8, and THAP11 regulons, as well as the known PPARG and CEBPE regulons (Fig. 5e) [22]. Subsequently, we used NicheNet analysis to investigate external regulators of the SPP1^+^ foamy macrophage phenotype [23]. We found that CSF1, which is produced in large quantities by mast cells, may function as a ligand to drive the phenotype of SPP1^+^ foamy macrophages (Fig. 5f). Mast cells were also more likely to interact with SPP1^+^ foamy macrophages according to the CellphoneDB, and CSF1–SIRPA interactions were among the most significantly enriched ligand-receptor combinations (Fig. 5g). Furthermore, we detected a significant increase in *CSF1* expression during the progression of atherosclerotic plaques by mining the bulk data (Fig. 5h, Additional file 5: Figure S5g). Besides, lesions were found to harbour a higher proportion of mast cells (Additional file 5: Figure S5g). A positive correlation was also observed between the proportion of mast cells and SPP1^+^ foamy macrophages in atherosclerotic lesions (Fig. 5i). Taken together, these results identify plausible candidate TFs that drove the phenotypic differences in SPP1^+^ foamy macrophages and indicate that the presence of CSF1^+^ mast cells may drive the phenotype of SPP1^+^ foamy macrophages in atherosclerosis.

### Observation of a cell-type-specific metabolic program, especially with macrophages

**Fig. 6:**
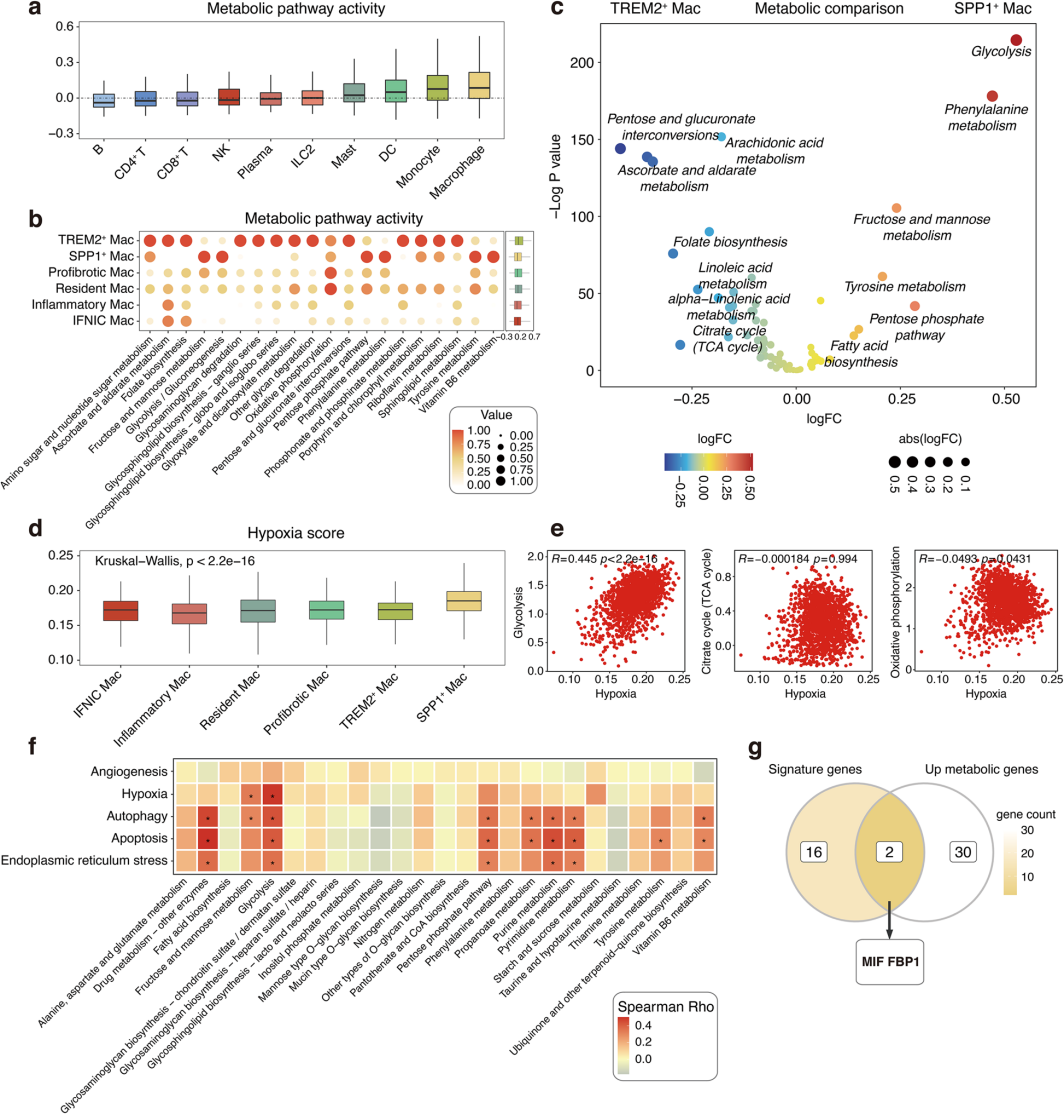
Metabolic heterogeneity of immune cell populations, especially macrophages. **a** Boxplot showing the metabolic pathway activity of the major immune cell populations. **b** Dotplot showing the activity of the top 20 variable metabolic pathways in the macrophage subpopulations (left). Boxplot showing the activity of all metabolic pathways in the macrophage subpopulations (right). **c** Volcano plot showing the differentially metabolic pathways between SPP1^+^ Mac and TREM2^+^ Mac. **d** Boxplot showing the hypoxic score of macrophage subpopulations. Kruskal–Wallis test. **e** Scatter plots comparing activities of glycolysis, citrate cycle, and oxidative phosphorylation response to hypoxia in SPP1^+^ Mac. Spearman rank test. **f** Heatmap showing the correlation between phenotypic score and metabolic activity score in SPP1^+^ Mac. *, *P* < 0.05 and Spearman rho > 0.3. Spearman rank test. **g** Venn diagram showing potential metabolic regulatory targets of SPP1^+^ Mac

Immunometabolism and the associated phenotypic biology in atherosclerotic disease remain unclear [24]. To understand the metabolic landscape of immune cells in plaques, the scores of all 77 active metabolic pathways were calculated using scMetabolism. Of all major cell types, myeloid cells consistently had the highest metabolic activity scores at both AC and PA sites (Fig. 6a, Additional file 6: Figure S6a). Interestingly, a comparison of pathway activities between AC and PA in the cell types shared by the two regions (excluding SPP1^+^ foamy macrophages) revealed high concordance between the counterparts (Additional file 6: Figure S6b).

Given that macrophages show the highest metabolic activity and play an important role in atherosclerotic lesions, we further explored the metabolic heterogeneity among different macrophage subpopulations. We selected the 20 most variable metabolic scores from among all subgroups and ordered macrophage clusters based on the average metabolic scores of all metabolic pathways (Fig. 6b). Intriguingly, TREM2^+^ foamy macrophages showed the greatest metabolic activity among all macrophages studied, whereas that of SPP1^+^ foamy macrophages were relatively low, which could be related to their specific functional differences in atherosclerotic plaques (Fig. 6b).

Further analysis of the differences in metabolic pathways between the two subpopulations revealed strong metabolic profiles associated with the cell type, identifying 25 metabolic pathways that were potentially upregulated in SPP1^+^ foamy macrophages and 52 that were upregulated in TREM2^+^ foamy macrophages (Fig. 6c). Among them, arachidonic acid, linoleic acid, and alpha-linolenic acid metabolism (the main sources of specialised pro-resolving mediators in plaques) were highly expressed in TREM2^+^ foamy macrophages, demonstrating the important role of these cells in orchestrating the resolution of tissue inflammation (Fig. 6c). In addition, variations in mitochondrial programs have also been found to be major contributors to cellular metabolic heterogeneity. In SPP1^+^ foamy macrophages, glycolysis has primacy over the citrate cycle, suggesting that these cells may require more rapid, short-term bursts of ATP production (Fig. 6c). Consistent with this possibility, the pentose phosphate pathway and fatty acid biosynthesis were also upregulated in SPP1^+^ foamy macrophages, whereas fatty acid degradation was upregulated in TREM2^+^ foamy macrophages (Fig. 6c). Previous findings have shown that metabolic reprogramming of the mitochondrial program to glycolysis may occur in the presence of hypoxia or normoxia, the latter being known as the Waberg effect [24]. Therefore, we next asked whether such highly state glycolysis metabolic activated states in SPP1^+^ foamy macrophages were induced by the Warburg effect. Surprisingly, the highest hypoxia score was observed for these cells (Fig. 6d). Indeed, the hypoxia-dependent HIF-1α signalling pathway was significantly enriched in SPP1^+^ foamy macrophages compared to that in other myeloid subtypes (Additional file 6: Figure S6c). In addition, we found that hypoxia correlated significantly with glycolysis, whereas the opposite was true for the mitochondrial program (Fig. 6e). In conclusion, our results indicate that SPP1^+^ foamy macrophages, undergoing endoplasmic reticulum stress caused by cholesterol overload, are more likely located in the hypoxic region of the AC, which jointly induces metabolic reprogramming of the mitochondrial programme.

Notably, some amino acid metabolism pathways, such as those for phenylalanine and tyrosine, were also significantly upregulated in SPP1^+^ foamy macrophages (Fig. 6c). Inhibition of such metabolic activity may mobilise dysfunctional efferocytosis to control the accumulation of oxidised low-density lipoproteins and apoptotic cells in advanced plaques. In addition, validation of these findings with a bulk data revealed that ruptured plaques showed upregulation of all energy-metabolism pathways (Additional file 6: Figure S6d), suggesting that they reflect the average expression levels over a mixture of different cell types, thereby masking the differences between cell types in the same sample and, in turn, demonstrating the importance of understanding the heterogeneity of cell metabolism at single-cell resolution.

To demonstrate the link between metabolic reprogramming and macrophage phenotypic changes, we further examined the relationship between the 25 upregulated metabolic pathways and phenotype scores of SPP1^+^ foamy macrophages. Several macrophage-specific metabolic processes were associated with autophagy, apoptosis, and endoplasmic reticulum stress (Fig. 6f). Analysis of the metabolic and marker genes upregulated in SPP1^+^ foamy macrophages revealed *MIF* (a critical enzyme for phenylalanine metabolism) and *FBP1* (a key enzyme in gluconeogenesis) as potential metabolic regulatory targets (Fig. 6g). The Enrichr was used to subsequently identify some potential drug candidates for these targets, including the well-known flavonol quercetin (Additional file 6: Figure S6e), which significantly reduce atherosclerotic plaque areas and lipid accumulation in the aorta of ApoE ^-/-^ mice [25]. Taken together, these findings reveal that gaining insight into the metabolic phenotypes of immune cells may help elucidate the mechanisms of plaque progression and establish future therapeutic strategies.

### Cell–cell communications between infiltrating immune cells and correlations between immune cell characteristics and patient survival

**Fig. 7:**
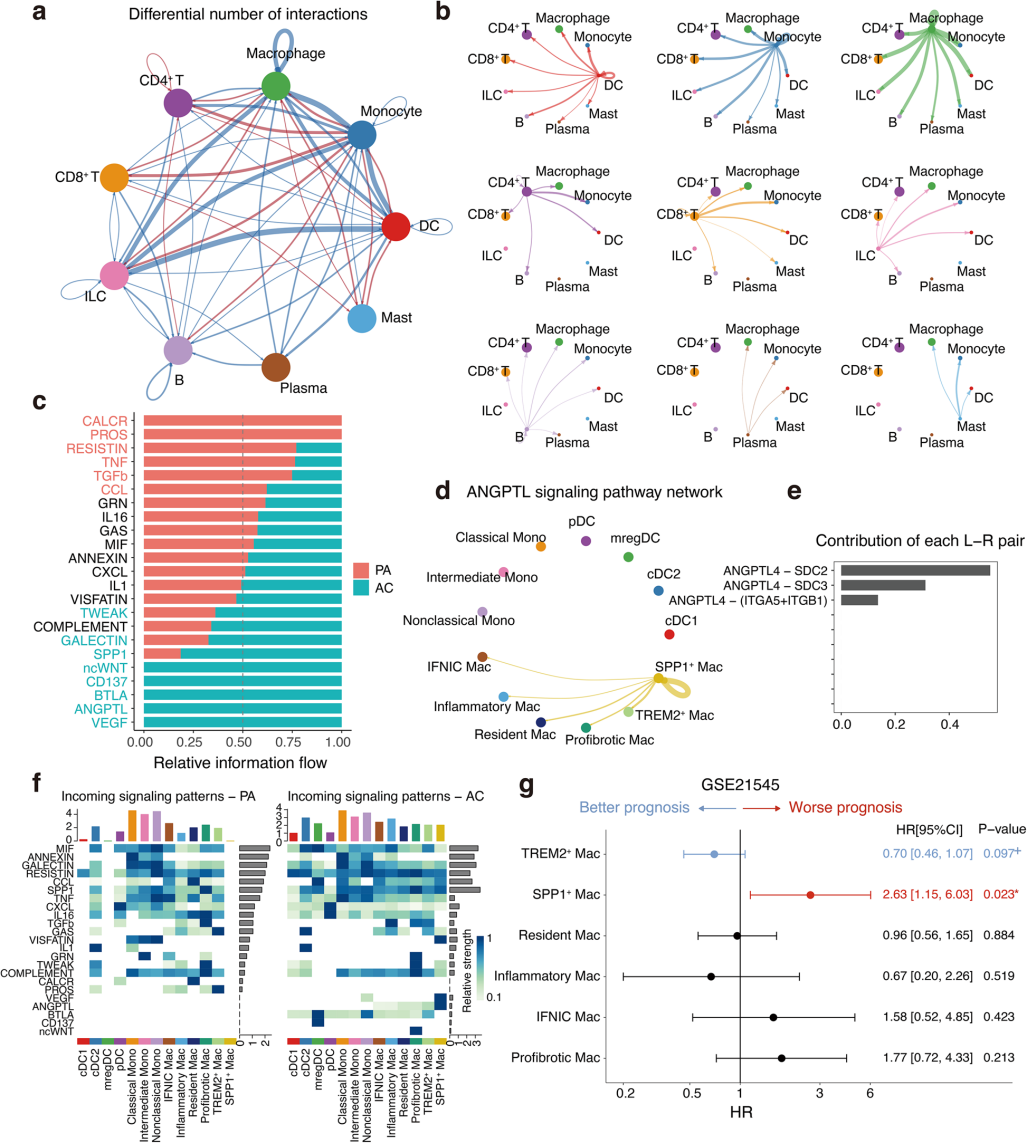
Cell–cell communications and prognostic analysis of major immune cell populations, especially macrophage subsets. **a** Circle plot showing the number of interactions between major immune cell types. Blue lines indicate that the displayed communication is decreased in AC, whereas red lines indicate that the displayed communication is increased in AC compared with PA. The thickness of the line is proportional to the number of unique ligand-receptor interactions, with loops representing autocrine circuits. **b** A detailed view of ligand and cognate receptor interaction for major immune cell types in the AC group. **c** Significant signaling pathways ranked based on differences in the overall information flow within the inferred networks between the PA and AC groups. Red, top pathways enriched in PA; black, equally enriched in PA and AC; green, enriched in AC. **d** The inferred ANGPTL signaling networks. Edge width represents the communication probability. **e** Relative contribution of each ligand-receptor pair to the overall communication network of ANGPTL signaling pathway. **f** Comparison of incoming signaling patterns of cells between the PA and AC groups. The colour is proportional to the contribution score computed from pattern recognition analysis. A higher score implies that the signaling pathway is more enriched in the corresponding cell group. **g** The correlation between estimated subpopulations and ischemic event (IE)–free survival in patients undergoing endarterectomy (*n* = 125). *P*-value was evaluated by the Cox proportional hazards model with 95% CI. *P* ≤ 0.05 were considered statistically significant in prognosis, whereas *p* > 0.05 and ≤ 0.1 were considered marginally significant in prognosis and represented as +

To characterise discrepancies in the molecular interactions between cells derived from different spatial sites of lesions, we utilised CellChat to construct a cell–cell-communication network based on known ligand-receptor pairs and their cofactors [26]. Interestingly, the PA region showed more intercellular interactions than the AC region, which was possibly due to reduced interactions between ILCs and myeloid cells and between myeloid and myeloid cells in the latter region (Fig. 7a, b).

Because of the high degree of communication between myeloid cells, we further visualised cellular communications between myeloid subpopulations. By comparing the overall information flow between the PA and AC, we identified 23 signalling pathways that were enriched in either the AC or PA, including a few other pathways were equally enriched in both regions (Fig. 7c). Notably, some pathways enriched in the AC have been implicated in the pathogenesis of atherosclerosis. For example, previous data showed that *ANGPTL4* was the most upregulated gene in foamy macrophages and that its deletion in haematopoietic cells led to a larger necrotic core and increased macrophage apoptosis [27]. We further demonstrated at the single-cell level that ANGPTL4-dependent signalling was sent from SPP1^+^ foamy macrophages to all macrophage subpopulations and that the signalling predominantly involved ANGPTL4–SDC2 interactions, among all known ligand-receptor pairs (Fig. 7d, e). We also performed a detailed analysis of changes in signalling-receptor levels for all important pathways. Some pathways were occurred active in AC cells, such as the ncWNT pathway that targets pro-fibrotic macrophages (Fig. 7f). In addition, VEGF signalling only targeted non-classical monocytes and SPP1^+^ foamy macrophages in AC (Fig. 7f). Some pathways were limited to cells in the PA, such as the CALCR signalling pathway that targets pDCs and resident macrophages, and the PROS signalling pathway that targets cDC2s and multiple macrophages (Fig. 7f). Some of the remaining pathways were found to vary with anatomical location. For example, IL1 signalling mainly targeted cDC2s and classical monocytes in the PA, whereas it only targeted cDC2s in the in AC (Fig. 7f). In addition, VISFATIN signalling only targeted monocytes in the in PA, whereas it only targeted SPP1^+^ foamy macrophages in the AC (Fig. 7f).

Finally, for validation purposes, we correlated eight major immune subtype-specific signatures using a larger patient cohort containing 223 samples from carotid plaques and peripheral blood mononuclear cells [28]. Based on the immune scores, these patients were subsequently divided into three groups, with blood being dominated by lymphocytes and plaques being dominated by macrophages and mast cells, which could be further divided into immune-activated and inactivated subgroups (Additional file 7: Figure S7a). Consistent with these observations, immune-related pathways were also upregulated in immune-activated plaques (Additional file 7: Figure S7b). However, survival analysis revealed no significant difference in the incidence of ischemic events after endarterectomy between the two groups, suggesting that further validation may be required (Additional file 7: Figure S7c). Then, we assessed the risk ratio for each macrophage subpopulation and patient population, as macrophages represent the main type of immune cells that infiltrate plaques. The results showed that TREM2^+^ foamy macrophage levels correlated with a better disease prognosis (Fig. 7g). Specifically, a higher abundance of SPP1^+^ foamy macrophages were significantly associated with a worse prognosis, which may be related to the dysfunction of these cells (Fig. 7g). Furthermore, MuSiC and Scaden, based on different deconvolution principles, also showed consistent results, further validating the important role of SPP1^+^ foamy macrophages in poor prognosis in atherosclerosis (Additional file 7: Figure S7d). In conclusion, clinical models for the stratification, survival prediction, and therapeutic evaluation of the immune microenvironment in patients with atherosclerosis are currently lacking [6]. In view of future anti-inflammatory therapies that specifically target plaques, it is imperative to establish reliable clinical models based on the interpretation of the plaque immune microenvironment enabled by single-cell sequencing.

## Discussion

**Fig. 8:**
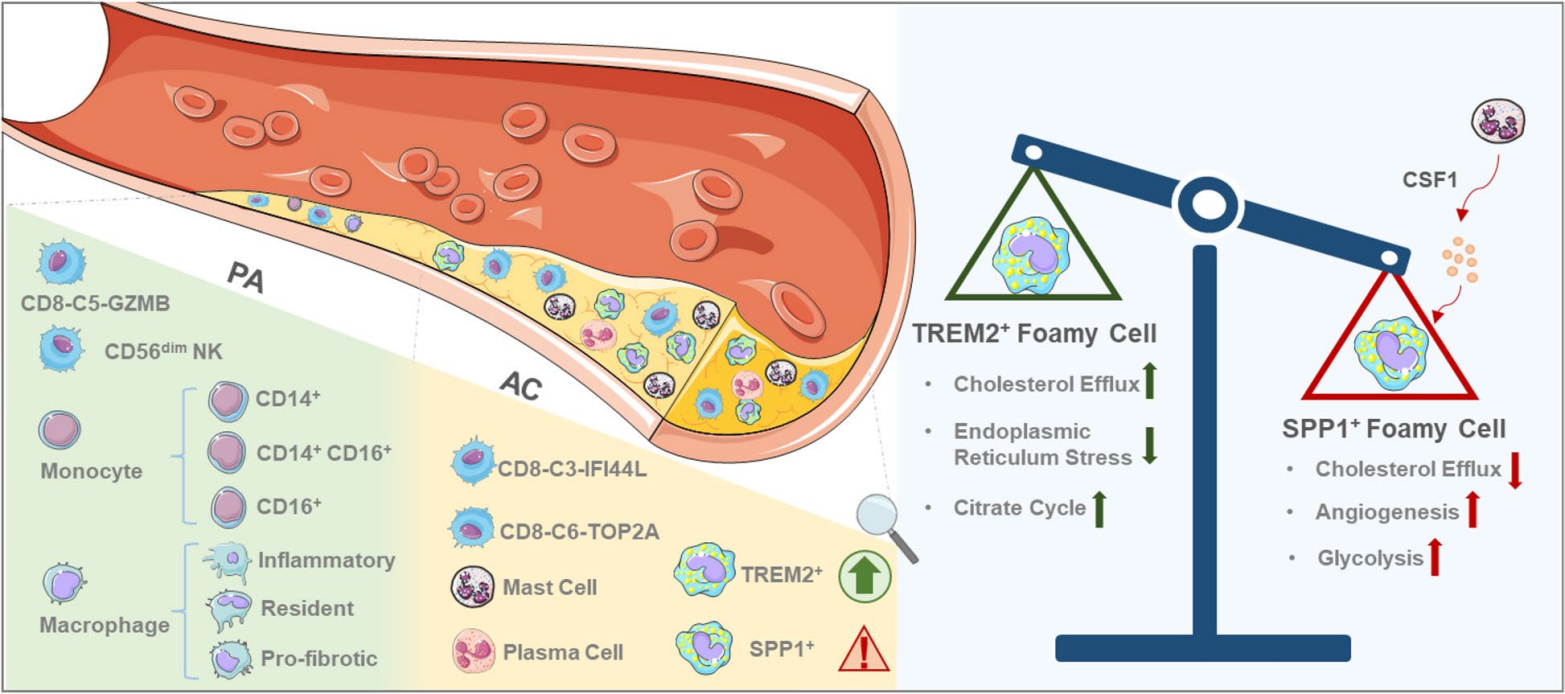
A model of the complex and heterogeneous immune microenvironment of atherosclerotic lesions

The uncertain and even contradictory results of the previous extensive anti-inflammatory treatments necessitates a more detailed characterisation of the complex immune microenvironment of atherosclerotic lesions and, thus, of locally targeted immunotherapies for plaques [2]. In this study, by integrating three human scRNA-seq datasets and using state-of-the-art analysis tools to overcome the limitations of a single study, we demonstrated that the atherosclerosis microenvironment is in fact more complex and heterogeneous than previously reported [7, 8]. Briefly, we analysed 44,120 cells from 17 human atherosclerosis samples and identified 28 different immune cell populations. Among these, we describe the functional properties and potential regulatory relationships of several cell subpopulations that have not been previously reported in similar studies, with an emphasis on TREM2^- ^SPP1^+^ foamy macrophages (Fig. 8). In addition, a landscape of cellular metabolisms and communications was constructed within the single-cell resolution, which will greatly contribute to the development of personalised diagnosis and treatment approaches.

Analysis of the integrated data revealed several previously uncharacterised T cell subsets. CD8-C3-IFI44L T cells expressed various interferon-stimulated genes, and interferon-response and virus-related pathways were enriched in these cells, suggesting that they served a proinflammatory role in plaques. Consistent with this possibility, type-I interferons within plaques can worsen atherogenesis [29]. Notably, previous data showed a potential causal relationship between viral infections and cardiovascular diseases [30]. A study by Chowdhury et al. further demonstrated the presence of numerous activated CD8^+^ T cells in plaques, which coincided with disease progression, and revealed that these cells were highly reactive to various viral antigens, including those from influenza, cytomegalovirus, EBV, and even SARS-CoV-2, suggesting that activation of these antigens by T cells could trigger a proinflammatory and soluble cell cascade, which in turn could increase mechanical stress and the size of the necrotic core in plaques [31]. Our current findings and these previous data help consolidate evidence for a potential causal relationship between viral infection and atherosclerosis, and further identify a potential cell population that may play an important role during the course of a viral response. In addition, these findings may highlight a potential candidate cell population for preventing and treating cardiovascular complications caused by various virus-related vaccines, including vaccines against SARS-CoV-2.

Fernandez et al. previously showed that T cells were more activated in the plaques of asymptomatic patients, whereas more exhausted T cells expressing PD-1 were found in the plaques of symptomatic patients [32]. Given that T cell activation exacerbates the progression of atherosclerosis, an urgent concern is that immunosuppressants such as PD-1 may have unanticipated effects in patients with underlying cardiovascular disease [33]. In contrast to their findings, we did not detect a clear exhausted phenotype in CD8^+^ T cells. However, we identified a population of cells expressing cell proliferation-, cytotoxicity-, and exhaustion-related genes, with a preference for distribution in the AC. CD8^+^ T cell exhaustion is currently a major obstacle to anti-tumour immunotherapies, and in-depth studies of these cells in cancer have defined a four-stage developmental framework: Tex^prog1^ (quiescent), Tex^prog2^ (proliferative), Tex^int^ (moderately cytotoxic), and Tex^term^ (terminally exhausted), and PD-1 pathway blockade has been shown to preferentially amplify Tex^prog2^ and Tex^int^ subpopulations [34]. Therefore, in conjunction with the results of that study, we believe that the group of cells in our study is more likely to resemble Tex^prog2^ cells or Tex^prog2^ cells just before transitioning to Tex^term^ cells. Therefore, it is worth mentioning that the exhausted T cells were previously described as a relatively homogenous population in plaques were possibly highly heterogeneous and displayed at least two different phenotypes, i.e., Tex^prog2^ and Tex^term^. It was also demonstrated that this activation-to-exhaustion transition and the clonal expansion of cells with both phenotypes is a continuous and gradual process under long-term inflammatory stimulation. However, the simultaneous presence of these subsets has not been observed in a single study of plaques to date, so we cannot help but question whether the different Tex phenotypes correlate with atherosclerosis severity. Furthermore, given the paradoxical nature of anti-cancer immunotherapy and autoimmune diseases, the stage where using immunosuppressants (such as PD-1) will minimise unnecessary cardiovascular toxicity in patients with cardiovascular diseases remains to be determined.

Link with Chowdhury et al.’s studies, we found that CD8-C5-GZMB may correspond to CD8 CTL Tem2, and CD8-C4-GZMK may correspond to CD8 CTL Tem1 [31]. Simultaneously, they also indicated that CD8 CTL Tem2 were most abundant in the complex plaque phenotype characterised by rupture, erosion, or thrombosis and least abundant in the calcified plaque phenotype, which is consistent with our Ro/e analysis. Notably, previous studies have also reported that KLRG1^+^ T effector cells may have developmental plasticity and that CX3CR1^+^ T effector cells are primarily a circulatory and vascular patrolling population [35–37]. In summary, integrating pseudotime analyses, cytoTRACE analyses, and migration molecule expression patterns, we revealed the possibility that more cytotoxic CD8^+^ T cells circulating in peripheral blood can home to lesions and differentiate with tissue-resident CD8^+^ T cells into less cytotoxic and more exhausted cells stimulated by a long-term chronic low-grade inflammatory plaque environment. Further detailed analysis of the immune dynamics of T cell development in atherosclerosis and the heterogeneity of exhausted T cells is needed in the future.

In this study, partially activated CD4^+^ T cells (CD4-C4-GAMA cells) showed high expression levels of cytokines and cytotoxic molecules, but lacked *CD28* expression, suggesting that these cells may be CD4^+^ CD28^null^ T cells previously identified in peripheral blood and plaques [16, 38]. Previous analyses of these cells have supported consensus views that a higher proportion of these cells are present in the peripheral blood of patients with acute coronary syndrome and that a significant positive correlation exist between their frequency and the number of cardiovascular events in patients with advanced atherosclerosis [39]. However, the results of one study revealed a positive association between the frequency of CD4^+^ CD28^null^ T cells and the first cardiovascular event in patients with diabetes, but without overt CVD [40]. In another population-based case–control cohort study, an increased frequency of these cells was associated with fewer first-time incidents of cardiovascular events [41].

These conflicting conclusions prompted us to conduct further research on CD4^+^ CD28^null^ T cells. Surprisingly, distinct differentiation fates were observed within these cells in this study, demonstrating that they do not comprise a homogeneous population. Cells with one differentiation fate in particular exhibited strong proinflammatory properties and significantly enriched lipids and atherosclerotic pathways. Apart from the pro-atherosclerotic features described above, these cells were also associated with the rate of post-endarterectomy ischemic events in patients with atherosclerosis. In summary, we believe that the above contradictory phenomena may be partly due to the fact that the homogenous CD4^+^ CD28^null^ T cells previously considered were essentially a relatively heterogeneous population, which confounds the interpretation of the variability between studies. Further in-depth studies based on the clarification of heterogeneity will help clarify the potential significance of these cells more accurately in cardiovascular disease.

Most notably, our findings paint a more refined and comprehensive picture of the immune landscape of myeloid cells, when compared to previous studies [7, 8]. For instance, at the single-cell level, we identified three well-defined monocyte subpopulations in human atherosclerotic plaques and analysed their functional heterogeneity. More importantly, we identified a previously unreported cell subpopulation. The high *SPP1* expression and low *TREM2* expression suggest that this group of cells does not belong to the previously described TREM2^+^ foamy macrophages [8, 18, 19]. The lower lysosome, cholesterol esterification, and cholesterol efflux scores, and higher endoplasmic reticulum stress, apoptosis, and autophagy scores (versus those of TREM2^+^ foamy macrophages) suggest that these cells may be dysfunctional foamy macrophages.

Comprehensive metabolic analysis at single-cell resolution revealed greater metabolic activities in myeloid cells, but also revealed unique metabolic heterogeneity in SPP1^+^ foamy macrophages, such as enhanced activity of glycolysis, pentose phosphate pathways, fatty acid biosynthesis, and phenylalanine- and tyrosine-metabolism pathways. Interestingly, in previous study, targeted metabolomics was used to demonstrate that high-risk carotid artery plaques obtained by endarterectomy exhibited elevated glycolysis, increased amino acid utilisation, and decreased fatty acid oxidation [42]. In addition, accumulation of the glucose analogue ^18^F-fluoro-2-deoxy-d-glucose (^18^F-FDG) in lesions was associated with macrophage-rich lesions that also showed increased glycolysis and pentose phosphate-pathway levels [43]. Based on these observations, ^18^F-FDG levels were measured in lesions by positron-emission tomography, which revealed progressive inflammatory lesions in humans and showed that increased ^18^F-FDG uptake correlated with an increased risk of cardiovascular disease [24, 44]. Unfortunately, the lack of methods for isolating cells and the subsequent analysis of macrophage metabolomes has hampered this research area. In this study, we drew upon the high-resolution capacity of scRNA-seq to resolve, for the first time, the metabolic heterogeneity of macrophages in plaques and revealed that SPP1^+^ foamy macrophages may correspond to the pathogenic cells that take up ^18^F-FDG in lesions, which have previously been identified at bulk levels.

Further studies showed that the increased glycolytic activity of SPP1^+^ foamy macrophages may be related to their location in the hypoxic region of the AC rather than Waberg effect [24]. Consistent with this phenomenon, SPP1^+^ foamy macrophages also exhibited upregulation of angiogenic pathways in response to hypoxic environments. The results of in vitro studies have confirmed that exposing macrophages to an anoxic environment can impair MerTK-mediated efferocytosis, which may be one of the causes of SPP1^+^ foamy macrophage dysfunction [45]. The interaction between mast cells co-located in the AC hypoxic region and SPP1^+^ foamy macrophages through CSF1–SIRPA pathways observed in this study may be another cause of the induced phenotype. Previous findings have shown that locally produced CSF1 derived from smooth muscle and endothelial cells appears to be the main driver of atherosclerosis, but the contribution of other cells, such as neutrophils, mast cells, and lymphocytes, to CSF1-dependent lesion growth requires further investigation [46].

Cell-communication analysis also revealed that different anatomical environments might shape different signal-communication patterns, such as ANGPTL, VEGF, and VISFATIN signals specific to SPP1^+^ foamy macrophages in the AC, some of which have been implicated in plaque progression [27, 47]. In addition, the presence of SPP1^+^ foamy macrophage signalling and the abundance of these cell subsets are significantly associated with the symptoms of disease progression in patients with atherosclerosis, including plaque rupturing, bleeding, and the recurrence of ischemic events after surgery. Therefore, we hypothesised that SPP1^+^ foamy macrophages may serve as key biomarkers of disease progression and targets for plaques in anti-inflammatory therapies.

This study had the following limitations: (1) Due to sample limitations, we only revealed a general disease-related immune heterogeneity without considering the heterogeneity of the diversity of risk factors, degree of disease progression, difference in vascular beds, and variability in treatment methods. (2) Global gene expression is merely an indirect method of assessing metabolism, and a thorough understanding of metabolism necessitates an understanding of metabolite concentrations and fluxes. (3) The loss of spatial information during cell dissociation leads to inadequate prediction of cell communication. Future single-cell multiomics studies with more elaborately designed experiments will further elucidate the pathogenesis of atherosclerosis.

## Conclusions

In conclusion, we further revealed the immune landscape in atherosclerotic plaques by integrating multiple datasets in an unbiased manner. These findings may provide insights into the function and regulation of atherosclerosis pathogenesis and progression, and should prove valuable in future immunotherapeutic strategies specifically targeting plaques.

## Methods

### Data acquisition

The scRNA-seq datasets were obtained from GSE131778 (https://www.ncbi.nlm.nih.gov/geo/query/acc.cgi?acc=GSE131778) [48], GSE155512 (https://www.ncbi.nlm.nih.gov/geo/query/acc.cgi?acc=GSE155512) [49] and GSE159677 (https://www.ncbi.nlm.nih.gov/geo/query/acc.cgi?acc=GSE159677) [50] in the Gene Expression Omnibus (GEO) database. Bulk mRNA arrays were also downloaded from GSE21545 (https://www.ncbi.nlm.nih.gov/geo/query/acc.cgi?acc=GSE21545) [28], GSE41571 (https://www.ncbi.nlm.nih.gov/geo/query/acc.cgi?acc=GSE41571) [51], GSE43292 (https://www.ncbi.nlm.nih.gov/geo/query/acc.cgi?acc=GSE43292) [52], GSE100927 (https://www.ncbi.nlm.nih.gov/geo/query/acc.cgi?acc=GSE100927) [53], and GSE163154 (https://www.ncbi.nlm.nih.gov/geo/query/acc.cgi?acc=GSE163154) [20].

### scRNA-seq data preprocessing and analysis

scRNA-seq datasets of human atherosclerotic plaques were downloaded from GEO database and re-analysed using Cellranger (v6.1.2) [54]. The raw gene-expression matrix was transformed into a Seurat object using the Seurat package (v4.0.5) of R (v4.1.1) [55]. In order to exclude low-quality cells, cells with greater than 25,000 unique molecular identifiers (UMIs) were removed and only expressing between 200 and 4500 genes and genes expressed in at least 3 cells were used for further analysis. The doublet cells were further identified and removed from the remaining cells by R package DoubletFinder (v2.0.3) [56]. After the above quality control, the integration workflow recommended by Seurat 4 (v4.0.5) is followed [57]. We identified the “anchors” in the different batches to construct a reference. First, we used the “SplitObject” function to divide the combined object into a list. Before finding the anchors, log-normalisation was performed, and 2000 highly variable genes (HGVs) were identified using the “vst” method. Next, we used the “FindIntegrationAnchors” function with default parameters to identify the anchors. The “IntegrateData” function, which returns a Seurat object with a batch-corrected expression matrix for all cells, was used to integrate the batches using the anchors. All of these cells were subsequently dimensionally reduced based on HGVs and top 20 principal components estimated by an Elbow plot. The data clustering was done using the graph-based clustering approach implemented in the Seurat package’s “FindNeighbor” function with the top 20 principal components and “FindClusters” function with the “resolution” parameter set to 0.5. The “RunTSNE” function was used for the visualisation plot with the two-dimensional t-distributed stochastic neighbour embedding (t-SNE) model, setting “dims” to 1:30. And known cell lineages were assigned to major cell clusters projected in the t-SNE model using well-known marker genes. Cell clusters were then manually assigned to the major cell types in accordance with these established markers. Any cluster that had multiple markers for two different cell types was manually eliminated as a doublet. The “FindAllMarkers” function with default parameters was used to list the markers of all cell populations. For subclustering of the major cell populations (CD45^+^ cells, myeloid cells and T/NK cells), the same procedure of finding HGVs, removing batch effects, dimensionality reduction, and clustering were repeated. Any cluster with an extraordinarily high number of detected genes or UMI count was manually discarded as a doublet.

### Tissue distribution of clusters

To quantify the tissue preference of each cluster, the observed to predicted cell number (Ro/e) ratio was calculated for each cluster in different tissues [9]. The chi-square test was used to determine the predicted cell numbers for each combination of cell clusters and tissues.

### Assessing the heterogeneity of single-cell populations

To examine the heterogeneity of main immune lineages in this study, ROGUE (v1.0), an entropy-based universal metric for assessing the purity of single-cell populations, was utilised with the default parameter settings for recommended pipelines [58]. As was reported by Liu et al., the ROGUE index has been adjusted to a range of 0 to 1. One denotes a perfectly pure subtype with no important genes, while zero denotes the population’s most heterogeneous condition.

### Developmental trajectory inference

The Monocle2 algorithm was used to explore the differentiation trajectories of the selected clusters [59]. The “subset” command of Seurat was used to separate the interesting cell clusters, and the “newCellDataSet” function of monocle2 was used to construct a CellDataSet object with the “lowerDetectionLimit” parameter set to 0.5. The low-quality cells and genes were then filtered using the “detectGenes” function and the “subset” function, respectively, with the “min_expr” parameter set to 0.1. This was done after computing size factors and estimating dispersions. The “differentialGeneTest” function was used to find differentially expressed genes among clusters along the trajectory. The “reduceDimension” function used the “DDRTree” method to reduce the dimensions. The functions “plot cell trajectory,” “plot genes in pseudotime,” and “plot genes branched heatmap” were used for visualisation after cell ordering. CytoTRACE (v0.3.3) analysis [60], an unsupervised framework for predicting relative differentiation states from single-cell transcriptomes, was also performed using the default settings of recommended pipelines to supplement the trajectory analysis. The functions “plotCytoGenes” and “plotCytoTRACE” were used for visualisation.

### SCENIC analysis

We used the Single-Cell Regulatory Network Inference and Clustering (SCENIC) approach to identify regulons, modules of one TF, its potential targets, and their activities [21]. Briefly, the workflow starts from the count matrix depicting the gene abundances for all cells and consists of three stages. First, co-expression modules are inferred using a regression per-target approach (GRNBoost2). Next, the indirect targets are pruned from these modules using cis-regulatory motif discovery (cisTarget). Lastly, the activity of these regulons is quantified via an enrichment score for the regulon’s target genes (AUCell) [61]. The SCENIC pipeline’s Python-based computational analysis tool, pySCENIC, was used in this study to analyse TF activity. Its command-line implementation, databases for cis-target (+ / − 10 kb from hg19-tss-centred-10 kb-7species.mc9nr), TF motifs (motifs-v9-nr.hgnc-m0.001-o0.0), and command-line options were used. Additionally, we used all 1839 TFs with motifs available in the motif database as input. Finally, the “CalcRSS” function was applied to identify the regulon with a high Regulon Specificity Score (RSS), and cell-type-specific regulon was obtained by RSS order [62].

### Cell–cell-communication analysis

NicheNet is an effective method for predicting the ligands triggering target cell transcriptome alterations [23]. We used NicheNet, which integrated gene expression data of cells from our Seurat object with a database of prior knowledge on signaling and gene regulatory networks, to discover potential ligands that may be responsible for the distinct phenotype of SPP1^+^ macrophages. We initially determined which genes were differentially expressed in SPP1^+^ macrophages, and we used the set of genes of interest to be those with log2FC > 0.5 and adjusted *P*-values < 0.05. All expressed genes in SPP1^+^ macrophages were used as a background of genes. When a gene has non-zero values in at least 10% of the cells of a cell type, it is deemed to be expressed. Additionally, SPP1^+^ macrophages were used to characterise sender cells and myeloid subpopulations to define receiver cells. The Pearson correlation coefficient between the ligand’s target predictions and the observed transcriptional response was used to rank the ligands expressed by one or more sender cells. Based on the NicheNet prebuilt prior model, which uses many curated ligand-receptor and signaling databases to infer interactions between sender ligands, receiver receptors, and downstream target genes, receiver cell receptors were deduced. CellPhoneDB was used to infer cell–cell interactions between SPP1^+^ macrophages and the remaining myeloid populations using the log-normalised expression data [63]. The parameters “iterations,” “threshold,” and “*p*-value” were set to 1000, 0.1, and 0.05, respectively. Based on the expression of ligand-receptor pairs, it was possible to determine the strength of the potential interaction between two cell groups. Based on a permutation test, the enriched ligand-receptor interactions between two cell groups were calculated. To comprehensively describe the cell–cell interactions, CellChat (v1.1.3) with the default settings of recommended pipelines was also used to infer the ligand-receptor pairs among all cell populations identified in this study [26]. CellChat makes predictions about the key signaling inputs and outputs for cells and how those cells and signals interact together to perform activities. CellChat uses diverse learning and quantitative contrasts to categorise signaling pathways and identify conserved and context-specific pathways. CellChat uses a law of mass action model to determine the communication likelihood of a ligand-receptor pair between two cell types. This model depends on the concentration of the ligand and receptor, any known cofactor concentrations, and the number of cells in each cell type. It is significant if the communication probability is statistically higher between these known cell types than between cell groupings that have been randomly permuted.

### Assessing the scores of different phenotypes

The signature genes of different phenotypes (cholesterol efflux, lysosome, endoplasmic reticulum stress, angiogenesis, phagocytosis, cholesterol esterification, apoptosis, autophagy, and HIF1A signal) were collected from the Molecular Signatures Database (MSigDB) [64]. The M1/M2 phenotype-related signature genes were obtained from Azizi, Elham et al. [65]. Genes associated with “classically activated” (M1) macrophages include *IL23, TNF, CXCL9, CXCL10, CXCL11, CD86, IL1A, IL1B, IL6, CCL5, IRF5, IRF1, CD40, IDO1, KYNU, CCR7,* while *IL4R, CCL4, CCL13, CCL20, CCL17, CCL18, CCL22, CCL24, LYVE1, VEGFA, VEGFB, VEGFC, VEGFD, EGF, CTSA, CTSB, CTSC, CTSD, TGFB1, TGFB2, TGFB3, MMP14, MMP19, MMP9, CLEC7A, WNT7B, FASL, TNFSF12, TNFSF8, CD276, VTCN1, MSR1, FN1,* and *IRF4* were used to define the signature of “alternatively activated” (M2) macrophages. And then, the AUCell algorithm with the default settings is used to infer phenotype-related score for selected cell populations with the AUCell package (v1.16.0) [21].

### Functional-enrichment analysis

To explore the functions of different cell types, the FindAllMarkers function was used to list the markers of all cell populations. Gene set enrichment analysis (GSEA) was used to assess the pathway-enrichment status [66]. To investigate the function of *TREM2*, related genes in different cell types were studied separately and the 30 genes with the highest GSEA scores were subjected to Gene Ontology (GO) function-annotation analysis. All enrichment analyses were performed using the clusterProfiler package (v4.2.2) [67]. Gene set variation analysis (GSVA) was used to analyse functional differences in the corresponding cell populations at different anatomical locations. Pathways with high differences in activity scores were selected using the limma package (v3.50.0) [68].

### Evaluation of metabolic activity at single-cell resolution

The metabolic activities of individual cells within each cell population were visualised and quantified using scMetabolism (v0.2.1), a recently established computational pipeline for quantifying metabolic activities in single cells [69]. The software uses a single-cell matrix file and the vision algorithm to calculate the activity score of each cell in each metabolic pathway. KEGG pathways and Reactome entries were pre-populated in the scMetabolism software. The altered data set was homogeneously transformed before being used to analyse metabolic activity. The Vision algorithm also determined the metabolic score. Finally, different groups’ metabolic activity of various pathways was assessed to identify pathways with significant differences. The KEGG metabolic gene sets were used for analysis in this study, with the method set to “VISION.” Then, “DotPlot.metabolism” and “BoxPlot.metabolism” functions were employed for visualisation.

### Bulk dataset preprocessing and analysis

The raw data were downloaded from the GEO database. It contains details on the platform, samples, and GSE records. The obtained raw gene-expression values were log2-transformed. According to the annotation information of the platform’s normalised data, probes were transformed into gene symbols. Moreover, the probes matching multiple genes were removed from these datasets. The final expression value was determined by averaging the gene expression values obtained from multiple probe measurements. Furthermore, the boxplot was used to evaluate the result of the data preprocessing. The heat maps and principal components plots were drawn to illustrate correlations between the different samples. Then, the limma package was used to identify differentially expressed genes, and the clusterProfiler package was used to perform all enrichment analyse [67, 68].

### Correlation and survival analyses with the gene expression signatures

To further investigate the clinical significance of identifying signature genes in this study, the expression of each gene signature was evaluated using single-sample gene set enrichment analysis (ssGSEA) [70]. CIBERSORTx was also used to construct a reference matrix to deconvolute the immune cell abundances of macrophage subpopulations in each patient [71]. In addition, CIBERSORTx results were verified by MuSiC and Scaden algorithms using the default settings of recommended pipelines based on different frameworks [72, 73]. To assess the prognostic values of the gene-expression signatures and macrophage subsets, the significance of the rate of ischemic events post-endarterectomy based on the ssGSEA score, MuSiC score, and Scaden score (binary: high vs. low) was evaluated by the two-sided log-rank test in the Survival package. And the optimum cut-off point for each score was determined based on the maximally selected log-rank statistics using the “surv_cutpoint” function of the “survminer” R package. In addition, hazard ratios (HRs) and adjusted *P*-values were obtained using age-adjusted Cox proportional risk models implemented in the R survival package.

### Statistical analysis

All statistical analyses were performed using R software (v4.1.1). All images were generated using R Studio. Student’s *t* test, Wilcoxon rank sum test, and Kruskal–Wallis test were used where indicated. *P*-values of > 0.05 were not considered statistically significant and are represented as n.s., and *p*-values of ≤ 0.05 are represented as follows: *p ≤ 0.05, **p ≤ 0.01, ***p ≤ 0.001, and ****p ≤ 0.0001. *P*-values were also adjusted based on the false discovery rate (FDR) for multiple-hypothesis testing in GSEA analysis.

## Supplementary Information

**Additional file 1: Figure S1. **Quality of data before and after integration and heterogeneity of major cell subpopulations. (a)Scatter plot showing comparison of data quality before (left) and after (right) integration. (b)t-SNE plot showing immune cells and non-immune cells from atherosclerosis lesions, color-coded by cell types. (c)Feature plots showing canonical marker genes, color-coded by expression levels. (d)t-SNE plots showing immune cells from atherosclerosis lesions, color-coded by the GSE ID (left) and Sample ID (right). (e)Heatmap showing the correlation between major immune cell populations. (f)Radar plot showing enrichment of GO term of B and Plasma cells.

**Additional file 2: Figure S2.** Expression of selected marker genes and functional annotation of selected cell populations. (a)t-SNE plots showing T and ILCs from atherosclerosis lesions, color-coded by the GSE ID (left) and Sample ID (right). (b)Violin plots showing the expression of selected marker genes for each cell type. (c)The GSEA hallmark pathways enriched in the CD8-C3-IFI44L subset. NES, normalized enrichment score. (d)Volcano plot showing differential gene expression for CD7-C1-KLRC1 and CD7-C2-FCGR3A subsets. Genes labeled have log-fold change > 1, Δ Percentage Difference > 30% and adjusted *P*-value from Wilcoxon rank sum test <0.05. (e)The GSEA KEGG pathways of the CD7-C1-KLRC1 and CD7-C2-FCGR3A populations.

**Additional file 3: Figure S3.** Trajectory analysis of CD8^+^ T populations. (a)Boxplot showing the differentiation potential of CD4^+ ^T subpopulations. CytoTRACE values are positively correlated with differentiation potential. (b)Boxplots showing the pseudotime and differentiation potential of CD8^+ ^T subpopulations. (c)Boxplots showing the functional status score of CD8^+^ T. (d)Dotplot showing the expression of genes associated with the migration of CD8^+ ^T subpopulations.

**Additional file 4: Figure S4.** Expression of selected marker genes and functional annotation of selected cell populations. (a)t-SNE plots showing myeloid cells from atherosclerosis lesions, color-coded by the GSE ID (left) and Sample ID (right). (b)Dotplot showing the expression of selected marker genes for dendritic cells and monocyte cells. DC, dendritic cells; Mono, monocyte cells. (c)Bar chart showing enrichment of GO term of DC-C1-CLEC9A and DC-C2-CD1C. (d)Heatmap showing the signatures of “mregDC” in different DC subsets. (e)Violin plots of Mac-C6-TREM2 and Mac-C7-SPP1 showing expression of myeloid lineage transcription factors *SPI1* and *CEBPB* and smooth muscle cell lineage transcription factors *MYOCD* and *MRTFA*. Mac, macrophage. (f)Boxplots showing the M1 and M2 signatures across all macrophage subsets.

**Additional file 5: Figure S5.** Expression of *TREM2* and characterization of SPPI^+^ Mac. (a)Violin plots showing the expression of *TREM2* in Mac-C5-CD9 and Mac-C6-TREM2. (b)Boxplots showing the expression of *TREM2* in atherosclerotic lesions (*n* = 29) and control arteries (*n* = 12) without atherosclerotic lesions (left) and paired early (*n* = 32) and advanced (*n* = 32) lesions (right). ****, *P* ≤ 0.0001. Wilcoxon rank sum test (left) and paired Student’s t test (right). (c)Summary of the correlation between 4 pathways with *TREM2*; Spearman Rho was shown in each square. (d)Boxplots showing phenotypic score of TREM2^+^ Mac and SPP1^+^ Mac. ****, *P* ≤ 0.0001. Wilcoxon rank sum test. (e)To establish a relationship between SPP1^+^ Mac and clinical disease, the enrichment of signature genes was tested on bulk data of macrophage-rich regions of stable (*n* = 5) and ruptured (*n* = 6) human plaques (top). Heatmap showing the leading genes of the SPP1^+^ Mac (bottom). (f)The developmental trajectory of monocyte and macrophage subsets, colored-coded by the associated cell subpopulations. (g)Boxplots showing the expression of *CSF1* (left) and infiltrating score of CSF1^+^ mast cells (right) in atherosclerotic lesions (*n* = 29) and control arteries (*n* = 12) without atherosclerotic lesions. *, *P* ≤ 0.05. Wilcoxon rank sum test (left) and Student’s t test (right).

**Additional file 6: Figure S6.** Metabolic characteristics of immune cell populations. (a)Boxplot showing the metabolic pathway activity of the major immune cell populations in PA (top) and AC (bottom). (b)Scatter plots comparing metabolic pathway activities between the PA and AC regions for myeloid subsets shared by the two regions. Spearman rank test. (c)t-SNE plots showing the enrichment score of HIF1α signal pathway. (d)Volcano plot showing the differentially metabolic pathways between ruptured and stable human plaques of bulk data GSE41571. (e)Enrichr analysis showing potential drug candidates targeting *MIF* or *FBP1*, sorted by combined score. Only the top 10 terms with *P* < 0.05 are shown.

**Additional file 7: Figure S7.** Patient immune infiltration stratification and prognostic analysis. (a)Heatmap showing patients were clustered into three groups, representing those with immune-activated (cluster 2 and 3) and immune-inactivated (cluster 1). (b)Immune-related pathways enriched in the plaque immune-activated cluster. (c)Kaplan–Maier survival curve of the ischemic event (IE)–free survival in patients undergoing endarterectomy stratified according to immune-activated plaque vs immune-inactivated plaque. Two-sided log-rank test. (d)Kaplan–Maier survival curve of the ischemic event (IE)–free survival in patients undergoing endarterectomy, stratified high and low according to the proportions of SPP1^+^ foamy macrophages.

## Data Availability

Publicly available datasets analysed during the current study are available in the GEO database under accession codes GSE131778 (https://www.ncbi.nlm.nih.gov/geo/query/acc.cgi?acc=GSE131778) [48], GSE155512 (https://www.ncbi.nlm.nih.gov/geo/query/acc.cgi?acc=GSE155512) [49], GSE159677 (https://www.ncbi.nlm.nih.gov/geo/query/acc.cgi?acc=GSE159677) [50], GSE21545 (https://www.ncbi.nlm.nih.gov/geo/query/acc.cgi?acc=GSE21545) [28], GSE41571 (https://www.ncbi.nlm.nih.gov/geo/query/acc.cgi?acc=GSE41571) [51], GSE43292 (https://www.ncbi.nlm.nih.gov/geo/query/acc.cgi?acc=GSE43292) [52], GSE100927 (https://www.ncbi.nlm.nih.gov/geo/query/acc.cgi?acc=GSE100927) [53], and GSE163154 (https://www.ncbi.nlm.nih.gov/geo/query/acc.cgi?acc=GSE163154) [20]. The core scripts to process and analyse data are available at https://github.com/jiexiong22/Plaque-CD45-scRNA [74].

## Abbreviations

LDL: Low-density lipoprotein
CVDs: Cardiovascular diseases
scRNA-seq: Single-cell RNA sequencing
HGVs: Highly variable genes
t-SNE: T-distributed stochastic neighbour embedding
TF: Transcription factor
GSEA: Gene set enrichment analysis
GO: Gene Ontology
GSVA: Gene set variation analysis
ssGSEA: Single-sample gene set enrichment analysis
ILCs: Innate lymphoid cells
AC: Atherosclerotic core
PA: Adjacent portion
IFN: Interferon
^18^F-FDG: ^18^F-fluoro-2-deoxy-d-glucose
