# Supplementary figures and images for: Bulk and single-cell characterisation of the immune heterogeneity of atherosclerosis identifies novel targets for immunotherapy
Source: BMC Biol. 2023 Feb 28;21:46. doi: 10.1186/s12915-023-01540-2 (PMC9974063; doi:10.1186/s12915-023-01540-2)

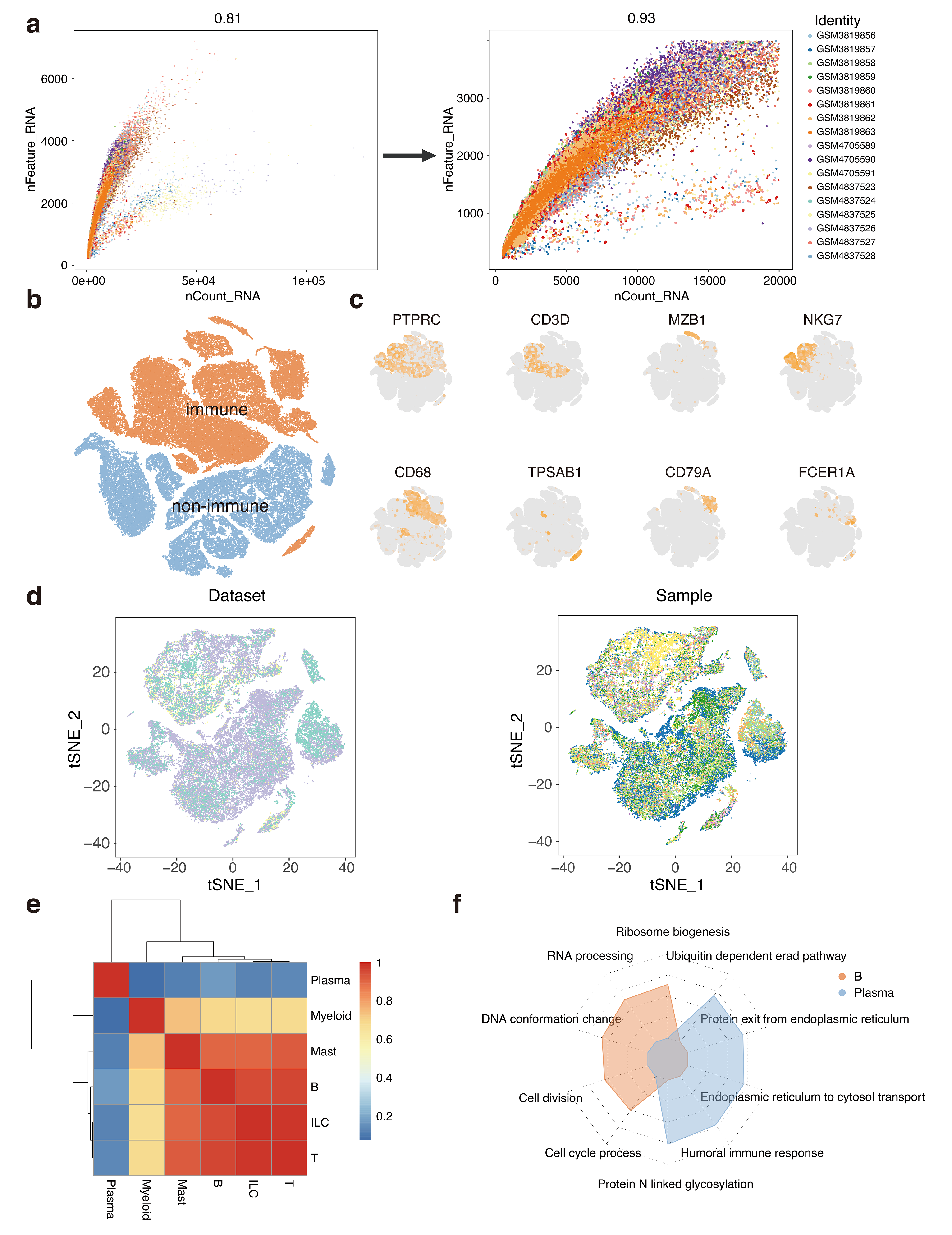

Supplement: Supplementary file 1 — Additional file 1: Figure S1. Quality of data before and after integration and heterogeneity of major cell subpopulations. (a)Scatter plot showing comparison of data quality before (left) and after (right) integration. (b)t-SNE plot showing immune cells and non-immune cells from atherosclerosis lesions, color-coded by cell types. (c)Feature plots showing canonical marker genes, color-coded by expression levels. (d)t-SNE plots showing immune cells from atherosclerosis lesions, color-coded by the GSE ID (left) and Sample ID (right). (e)Heatmap showing the correlation between major immune cell populations. (f)Radar plot showing enrichment of GO term of B and Plasma cells. [file 12915_2023_1540_MOESM1_ESM.tif]

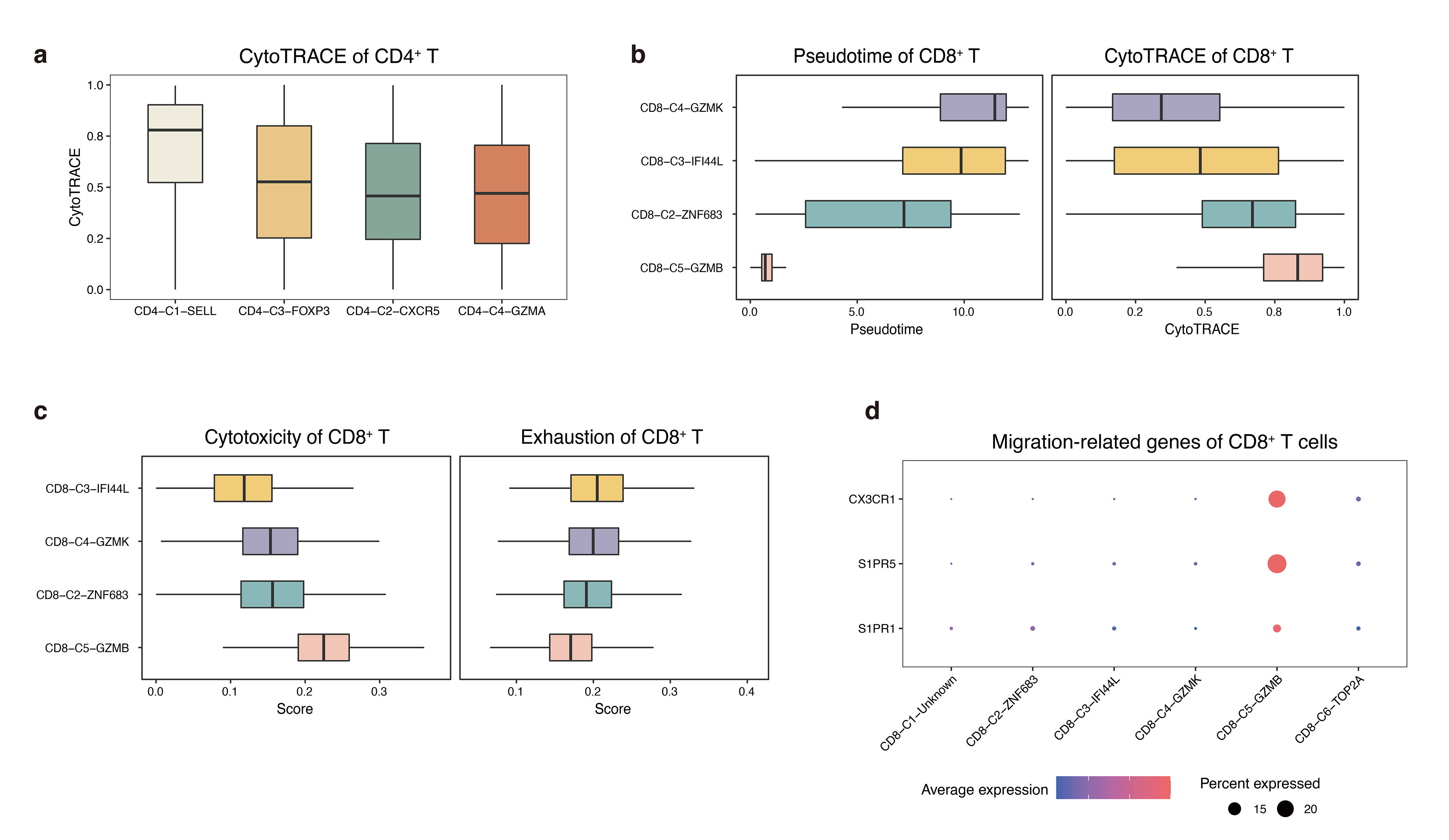

Supplement: Supplementary file 3 — Additional file 3: Figure S3. Trajectory analysis of CD8+ T populations. (a)Boxplot showing the differentiation potential of CD4+ T subpopulations. CytoTRACE values are positively correlated with differentiation potential. (b)Boxplots showing the pseudotime and differentiation potential of CD8+ T subpopulations. (c)Boxplots showing the functional status score of CD8+ T. (d)Dotplot showing the expression of genes associated with the migration of CD8+ T subpopulations. [file 12915_2023_1540_MOESM3_ESM.tif]

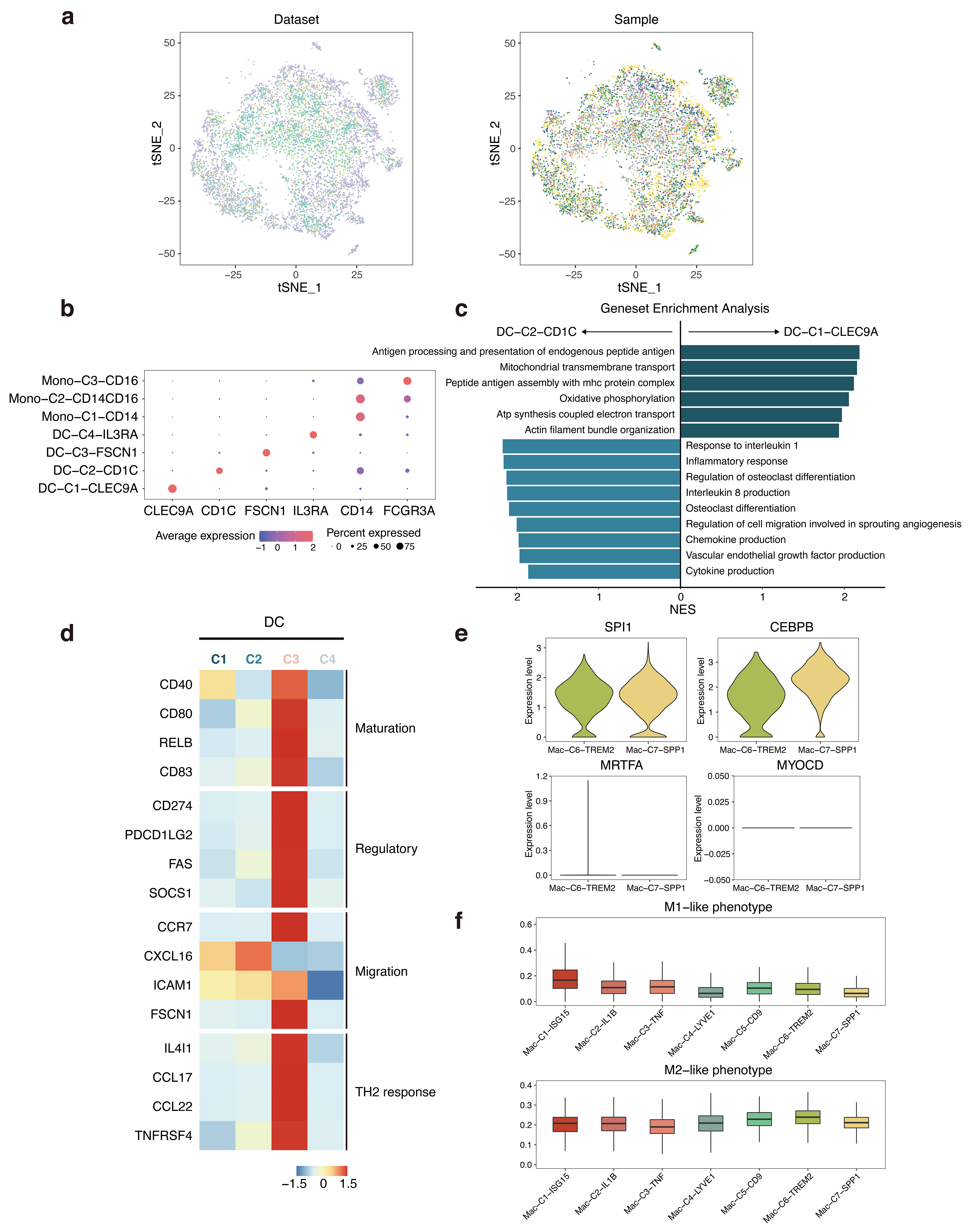

Supplement: Supplementary file 4 — Additional file 4: Figure S4. Expression of selected marker genes and functional annotation of selected cell populations. (a)t-SNE plots showing myeloid cells from atherosclerosis lesions, color-coded by the GSE ID (left) and Sample ID (right). (b)Dotplot showing the expression of selected marker genes for dendritic cells and monocyte cells. DC, dendritic cells; Mono, monocyte cells. (c)Bar chart showing enrichment of GO term of DC-C1-CLEC9A and DC-C2-CD1C. (d)Heatmap showing the signatures of “mregDC” in different DC subsets. (e)Violin plots of Mac-C6-TREM2 and Mac-C7-SPP1 showing expression of myeloid lineage transcription factors SPI1 and CEBPB and smooth muscle cell lineage transcription factors MYOCD and MRTFA. Mac, macrophage. (f)Boxplots showing the M1 and M2 signatures across all macrophage subsets. [file 12915_2023_1540_MOESM4_ESM.tif]

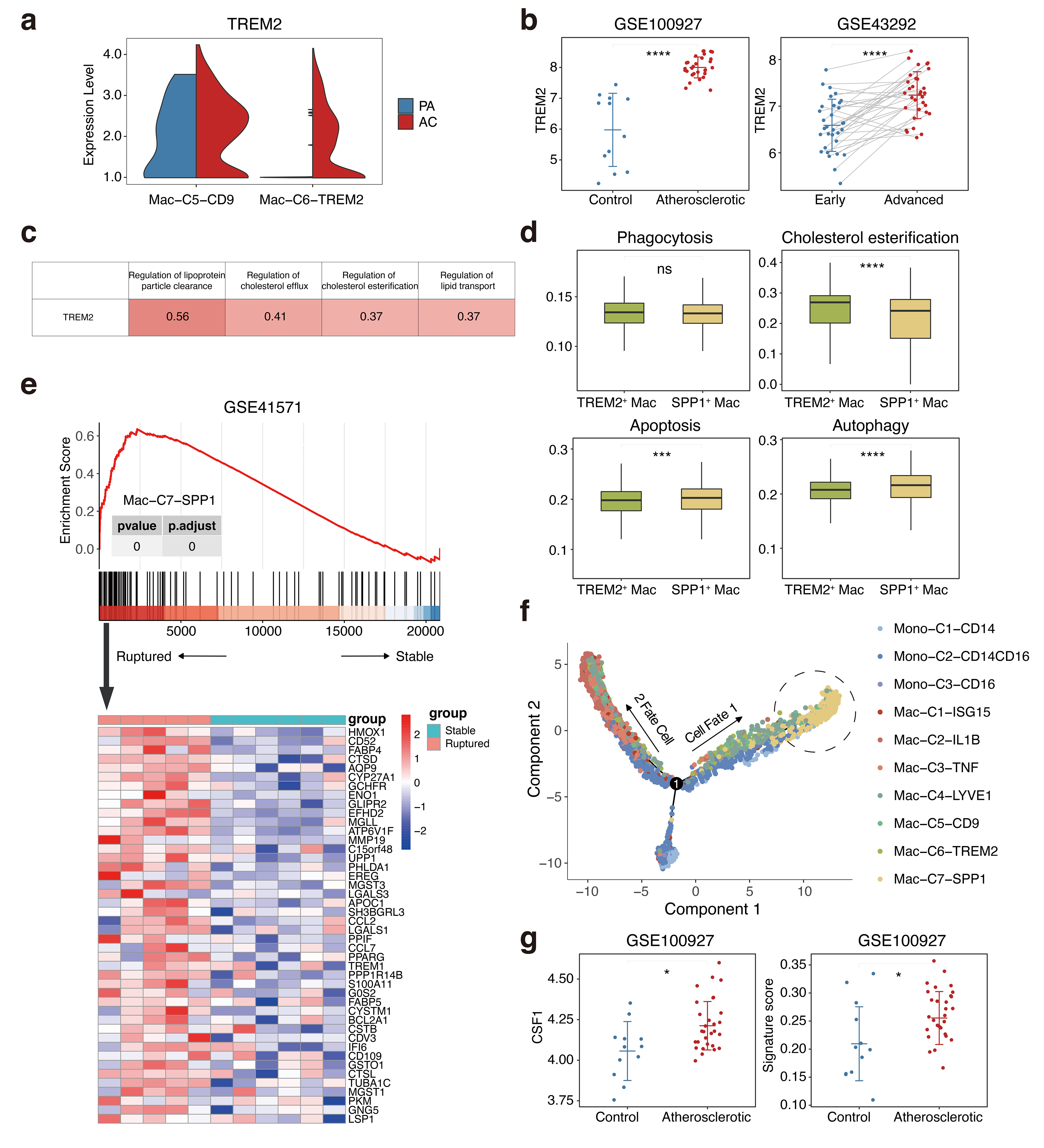

Supplement: Supplementary file 5 — Additional file 5: Figure S5. Expression of TREM2 and characterization of SPPI+ Mac. (a)Violin plots showing the expression of TREM2 in Mac-C5-CD9 and Mac-C6-TREM2. (b)Boxplots showing the expression of TREM2 in atherosclerotic lesions (n = 29) and control arteries (n = 12) without atherosclerotic lesions (left) and paired early (n = 32) and advanced (n = 32) lesions (right). ****, P ≤ 0.0001. Wilcoxon rank sum test (left) and paired Student’s t test (right). (c)Summary of the correlation between 4 pathways with TREM2; Spearman Rho was shown in each square. (d)Boxplots showing phenotypic score of TREM2+ Mac and SPP1+ Mac. ****, P ≤ 0.0001. Wilcoxon rank sum test. (e)To establish a relationship between SPP1+ Mac and clinical disease, the enrichment of signature genes was tested on bulk data of macrophage-rich regions of stable (n = 5) and ruptured (n = 6) human plaques (top). Heatmap showing the leading genes of the SPP1+ Mac (bottom). (f)The developmental trajectory of monocyte and macrophage subsets, colored-coded by the associated cell subpopulations. (g)Boxplots showing the expression of CSF1 (left) and infiltrating score of CSF1+ mast cells (right) in atherosclerotic lesions (n = 29) and control arteries (n = 12) without atherosclerotic lesions. *, P ≤ 0.05. Wilcoxon rank sum test (left) and Student’s t test (right). [file 12915_2023_1540_MOESM5_ESM.tif]

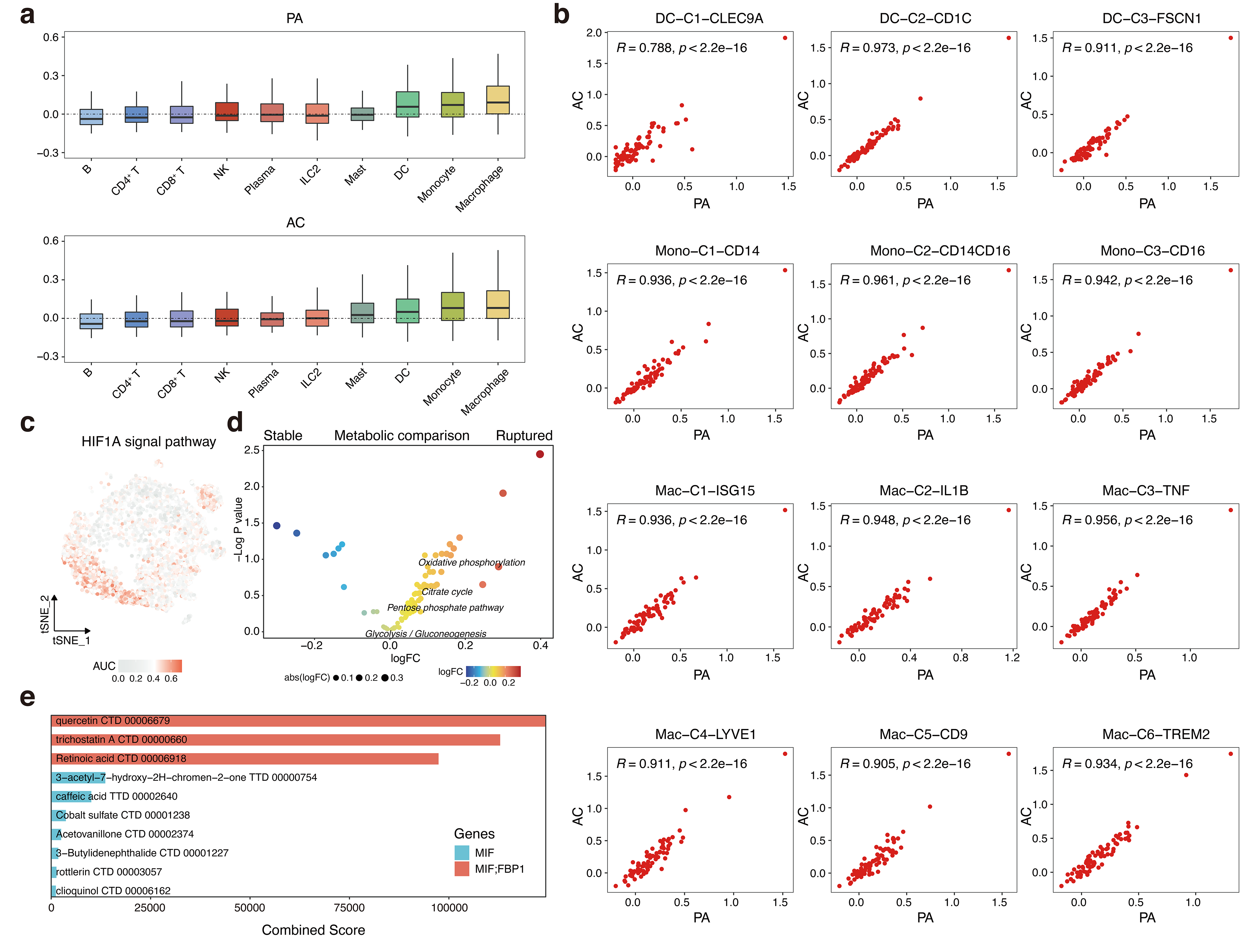

Supplement: Supplementary file 6 — Additional file 6: Figure S6. Metabolic characteristics of immune cell populations. (a)Boxplot showing the metabolic pathway activity of the major immune cell populations in PA (top) and AC (bottom). (b)Scatter plots comparing metabolic pathway activities between the PA and AC regions for myeloid subsets shared by the two regions. Spearman rank test. (c)t-SNE plots showing the enrichment score of HIF1α signal pathway. (d)Volcano plot showing the differentially metabolic pathways between ruptured and stable human plaques of bulk data GSE41571. (e)Enrichr analysis showing potential drug candidates targeting MIF or FBP1, sorted by combined score. Only the top 10 terms with P < 0.05 are shown. [file 12915_2023_1540_MOESM6_ESM.tif]

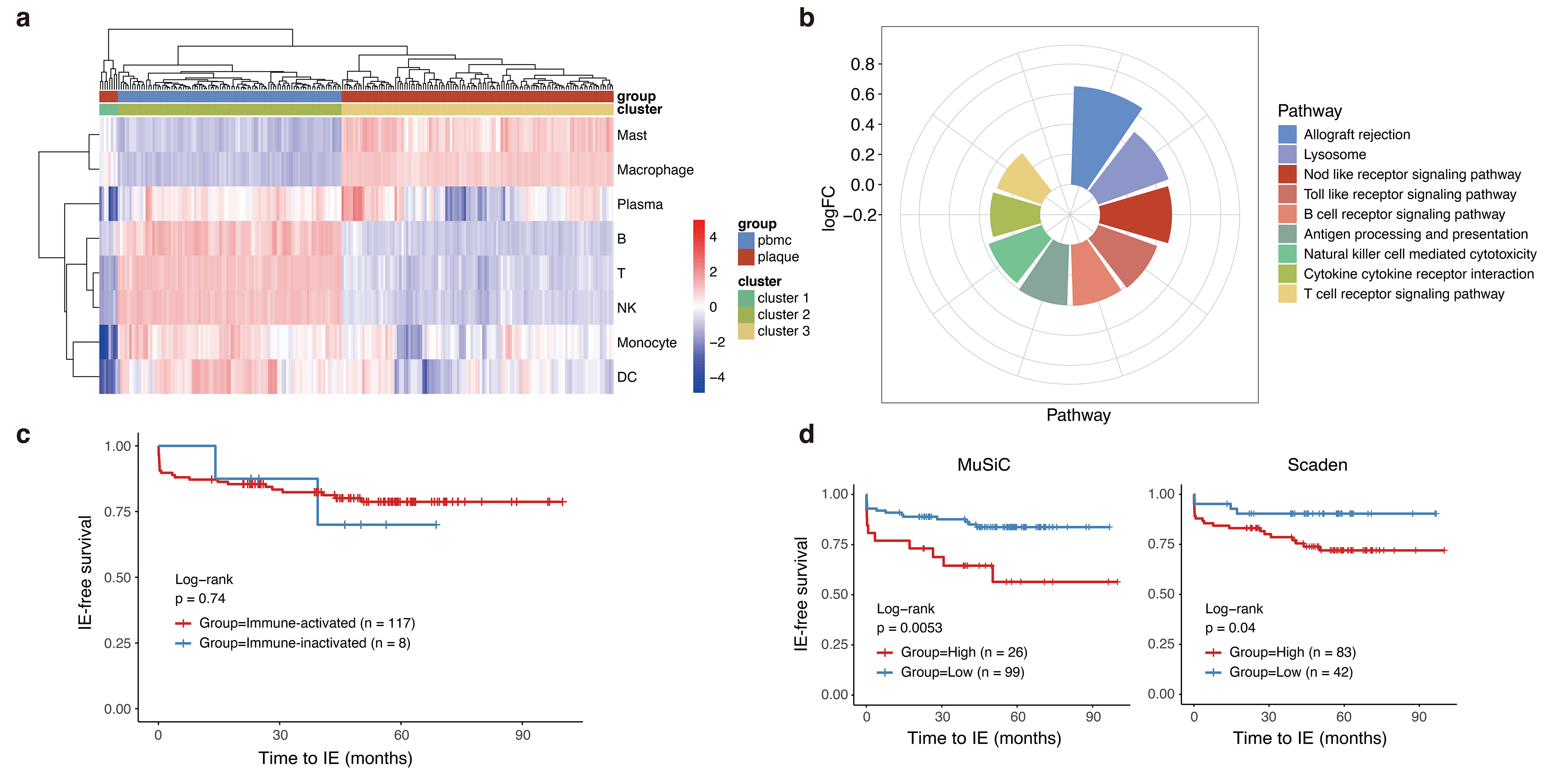

Supplement: Supplementary file 7 — Additional file 7: Figure S7. Patient immune infiltration stratification and prognostic analysis. (a)Heatmap showing patients were clustered into three groups, representing those with immune-activated (cluster 2 and 3) and immune-inactivated (cluster 1). (b)Immune-related pathways enriched in the plaque immune-activated cluster. (c)Kaplan–Maier survival curve of the ischemic event (IE)–free survival in patients undergoing endarterectomy stratified according to immune-activated plaque vs immune-inactivated plaque. Two-sided log-rank test. (d)Kaplan–Maier survival curve of the ischemic event (IE)–free survival in patients undergoing endarterectomy, stratified high and low according to the proportions of SPP1+ foamy macrophages. [file 12915_2023_1540_MOESM7_ESM.tif]
